# Environmental Mold and Mycotoxin Exposures Elicit Specific Cytokine and Chemokine Responses

**DOI:** 10.1371/journal.pone.0126926

**Published:** 2015-05-26

**Authors:** Jamie H. Rosenblum Lichtenstein, Yi-Hsiang Hsu, Igor M. Gavin, Thomas C. Donaghey, Ramon M. Molina, Khristy J. Thompson, Chih-Lin Chi, Bruce S. Gillis, Joseph D. Brain

**Affiliations:** 1 Molecular and Integrative Physiological Sciences Program, Department of Environmental Health, Harvard T.H. Chan School of Public Health, Boston, MA, United States of America; 2 Institute for Liberal Arts, Emerson College, Boston, MA, United States of America; 3 Hebrew Senior Life Institute for Aging Research and Harvard Medical School, Boston, MA, United States of America; 4 Broad Institute of MIT and Harvard, Cambridge, MA, United States of America; 5 Research Resources Center, University of Illinois at Chicago, Chicago, IL, United States of America; 6 Institute for Health Informatics, University of Minnesota School of Nursing, Minneapolis, MN, United States of America; 7 Department of Medicine, University of Illinois College of Medicine, Chicago, IL, United States of America; University of Leuven, Rega Institute, BELGIUM

## Abstract

**Background:**

Molds can cause respiratory symptoms and asthma. We sought to use isolated peripheral blood mononuclear cells (PBMCs) to understand changes in cytokine and chemokine levels in response to mold and mycotoxin exposures and to link these levels with respiratory symptoms in humans. We did this by utilizing an *ex vivo* assay approach to differentiate mold-exposed patients and unexposed controls. While circulating plasma chemokine and cytokine levels from these two groups might be similar, we hypothesized that by challenging their isolated white blood cells with mold or mold extracts, we would see a differential chemokine and cytokine release.

**Methods and Findings:**

Peripheral blood mononuclear cells (PBMCs) were isolated from blood from 33 patients with a history of mold exposures and from 17 controls. Cultured PBMCs were incubated with the most prominent *Stachybotrys chartarum* mycotoxin, satratoxin G, or with aqueous mold extract, ionomycin, or media, each with or without PMA. Additional PBMCs were exposed to spores of *Aspergillus niger*, *Cladosporium herbarum* and *Penicillium chrysogenum*. After 18 hours, cytokines and chemokines released into the culture medium were measured by multiplex assay. Clinical histories, physical examinations and pulmonary function tests were also conducted. After *ex vivo* PBMC exposures to molds or mycotoxins, the chemokine and cytokine profiles from patients with a history of mold exposure were significantly different from those of unexposed controls. In contrast, biomarker profiles from cells exposed to media alone showed no difference between the patients and controls.

**Conclusions:**

These findings demonstrate that chronic mold exposures induced changes in inflammatory and immune system responses to specific mold and mycotoxin challenges. These responses can differentiate mold-exposed patients from unexposed controls. This strategy may be a powerful approach to document immune system responsiveness to molds and other inflammation-inducing environmental agents.

## Introduction

Indoor environments contaminated by molds cause adverse human health effects [1]. Cellulose, when combined with moisture and warm temperatures, promotes mold growth. Chronic mold exposures at home, work or school are associated with increased upper and lower respiratory symptoms [2]. This is commonly attributed to an allergen-dependent pathway, but there is evidence that mold may also trigger asthma in an allergen-independent manner [3, 4]. Mold and mold-related odors are an important cause of atopic symptoms, allergic sensitization and asthma [5]. Mold exposures have been shown to cause a nine-fold increase in emergency room visits for asthma symptoms among asthmatics [6]. Mold exposures may increase sensitivity to commonly inhaled microorganisms and inert substances and increase risks of secondary infections [7].

Both mold hyphae and conidia induce immune responses in humans [8]; however, no reliable tests linking clinical symptoms with exposures have been reported. Most clinical studies have used self-reported symptoms and were based on subjective complaints prone to bias and confounders [9, 10]. Existing clinical tests also fail to establish a definitive link between chronic mold exposures and adverse health effects. More reliable mold-related tests are needed [11]. Common immunological tests, such as IgE measurements (RAST analysis) or skin prick tests, are poor indicators of mold exposure [12, 13].

We looked for immune system modulators that might link molds and mold-related substances with respiratory illness in humans. The mold *Stachybotrys chartarum* (*S*. *chartarum*) can be found in both indoor and outdoor environments [14]. Modern building materials, such as cellulose-based wallboard and ceiling tiles, have made *S*. *chartarum* more common in indoor environments [15]. Severe weather such as Hurricane Katrina can cause water intrusions in homes, offices and schools; the resulting wet substrates foster the growth of *S*. *chartarum* [16, 17]. Nevertheless, there are limited studies of humans focusing on inflammatory responses [18] or biomarkers of exposures [19] to *S*. *chartarum* [20].

*S*. *chartarum* causes symptoms such as runny nose, cough, headache, and asthma exacerbations [21]. Several studies correlated infant idiopathic pulmonary hemorrhage with *S*. *chartarum* exposures in homes [22–25], though a subsequent CDC report [26] noted some flaws in the initial reports. There exists strong evidence that *S*. *chartarum* causes acute inflammatory responses, macrophage cytotoxicity [27], pulmonary hemorrhage [28], lung inflammation [29, 30] and asthma-like responses [31] in mice. *S*. *chartarum* has also been correlated with asthma in children exposed at school [32]. Acute responses to *S*. *chartarum* are usually associated with mycotoxins, including the trichothecene satratoxin G (SG), which interferes with RNA synthesis and leads to apoptosis [33–35]. We have shown that *Stachybotrys* spore toxin (SST), a mixture of mycotoxins, causes pulmonary hemorrhage as well as cytokine and chemokine production in a murine model [27].

To better understand the human health risks associated with mold exposures and to identify strategies to document health consequences from indoor mold exposures, we studied responses of peripheral blood mononuclear cells (PBMCs) from individuals who were exposed to molds in their workplaces. We hypothesized that chronic exposures to molds may induce tolerance and/or sensitization to these allergens, thereby decreasing some allergen-specific immune responses while increasing others. Mouse models show that chronic allergen exposure sometimes creates both tolerance, marked by suppression of some inflammatory responses such as eosinophilia, and sensitization, marked by GM-CSF expression and dendritic and CD4+ T-cell activation [36].

Cytokines and chemokines are mediators of inflammatory and immune system responses. Many macrophage and epithelial cell lines produce cytokines and chemokines after challenges to mold spores or hyphae [28, 37–39]. These facts provided additional rationale for this study. Since tolerance and sensitization are induced at the cellular level [40], we hypothesized that repeated mold exposures might cause specific persistent alterations in the expression of cytokines and chemokines when isolated PBMCs respond to subsequent exposures to specific molds, their mycotoxins, or other immune challenges. The pattern of altered expression of these cytokines and chemokines might then represent a signature of the mold exposure. To test this hypothesis, we isolated PBMCs from mold-exposed patients and unexposed controls. We then exposed those PBMCs to mold or mycotoxins *ex vivo* and compared their immunologic and inflammatory responses. We also subdivided the mold-exposed patients into asthmatics and nonasthmatics to explore whether any subset of cytokines and chemokines might distinguish them.

## Materials and Methods

We investigated (1) if PBMCs respond to mold and/or mycotoxin challenges by altering cytokine and chemokine production, and (2) if the magnitude of any response was affected by previous chronic exposures to mold.

### Ethics Statement

Informed signed written consent for blood collection and testing was obtained from all participants using a form and procedures approved by the Institutional Review Board Services, Aurora, Ontario, Canada (FDA/OHRP IORG Registration # IORG0000456). The Harvard T.H. Chan School of Public Health Office of Human Research Administration approved the *ex vivo* mycotoxin exposures and associated data collection.

### Human Mold Exposures

Industrial hygiene consultants (Public Health and Safety, Irvine, CA) tested two office buildings with water intrusions in the Los Angeles metro area. They reported detectable levels of culturable molds. The industrial hygiene testing was approved by and performed according to the guidelines of the State of California Department of Workers’ Compensation. Air, surface and material samples collected from 37 sites throughout the two affected buildings, including multiple portions of their ventilation systems, were tested for viable mold. The fungal colonies were cultured and identified by microscopic and macroscopic morphology.

### Patient and Control Selection

All 33 patients in this study worked in one of the affected office settings. The primary jobsites of mold-exposed patients were spread throughout different parts of these two buildings. All of the mold-exposed patients had occupational exposures from 1–5 years in length to *S*. *chartarum*, *A*. *niger*, *C*. *herbarum*, and *P*. *chrysogenum*. None reported experiencing water intrusions and/or mold contamination in any other personal environments, such as their residences. The mold-exposed patients did not complain of chronic respiratory symptoms until working in the contaminated buildings and had subsequently sought medical evaluations. The onset of symptoms varied from 1–3 months to over a year after starting in these job environments. Twenty-nine mold-exposed patients had blood samples collected while they were working in a contaminated building. Four mold-exposed patients had blood samples collected between 6 months and 2 years after transferring to a different work environment. We collected additional blood samples from 15 of the mold-exposed patients 18 months after the initial collection to assess the stability of responses. We do not know how often or how intensely the patients were exposed to the molds, spores, glucans, and mycotoxins detailed in the industrial hygiene reports.

The control groups were healthy adults from the Los Angeles metro area who did not work in the same buildings. The control subjects had no known history of exposures to molds at work or home and they lacked any related symptoms. None of the control subjects complained of chronic respiratory symptoms nor did they possess any medical history of chronic respiratory ailments. The controls for the mycotoxin experiments were on average slightly younger and had a different ethnic composition than the mold-exposed patients. The gender distribution was similar between the mold-exposed patients and controls.

These experiments included a total of 33 mold-exposed patients. Cells from 27 mold-exposed patients and 17 unexposed control subjects were challenged with *S*. *chartarum* mycotoxins *ex vivo*. Cells from five of these mold-exposed patients, together with six additional mold-exposed patients and eleven additional unexposed controls, comprising a “mold spore group,” were also challenged with spores from three additional molds. Demographic information, including age, gender, ethnicity, smoking status, pulmonary function and BMI, was collected from the mold-exposed patients.

### Mold Spores and Mycotoxins

For immunologic challenges of cultured PBMCs with molds, the following mold strains were purchased from the American Tissue Culture Collection (Manassas, VA): *S*. *chartarum*, ATCC201212; *A*. *niger*, ATCC16888; *C*. *herbarum*, ATCC11281; and *P*. *chrysogenum*, ATCC10106.

The *S*. *chartarum* strain used was an ATCC clone originally isolated from an affected home during a Cleveland outbreak of infant idiopathic pulmonary hemorrhage [41]. This strain was chosen because it is well characterized and expresses a range of mycotoxins: satratoxin G (SG), satratoxin H, stachylysin, trichoverrol B, and roridin L-2 [41].

*S*. *chartarum* spores were grown on potato dextrose agar (PDA) plates at 15°C. After approximately 35 days of growth, spores were dried for 21 days and vacuumed using a modified filter cassette with a 37-mm, 0.4-μm polycarbonate membrane filter (Poretics Corp., Livermore, CA). These methods are modified from Rao *et al*. [42]. Dried spores were stored in glass at room temperature. We prepared *Stachybotrys* Spore Toxin (SST) from the *S*. *chartarum* spores, as previously described [27]. SST is a mixture of mycotoxins and other water-soluble extracts [27]. SG was obtained from Dr. James Pestka (Michigan State University, East Lansing, MI) [43]. Both toxin preparations were suspended in D-PBS and sterile filtered at 0.1 μm (Acrodisc syringe filters, Pall Life Sciences, Port Washington, NY). SG was diluted 1:1000 in media to a final concentration of 14.3 ng/ml. SST concentrations were standardized among batches based on SG content to a final SG concentration of 26.4 ng/ml in media. These toxin concentrations were determined in a small dose-response pilot study to cause <1% cytotoxicity.

Spore samples of *A*. *niger*, *C*. *herbarum*, and *P*. *chrysogenum* were suspended to a total volume of 1.4 ml in phosphate buffered saline (PBS) to final concentrations of 800 and 4,000 spores/ml (*A*. *niger)*, 2,000 and 10,000 spores/ml (*C*. *herbarum)*, and 4,000 and 20,000 spores/ml (*P*. *chrysogenum)*. Spore concentrations were determined by counting spores in a counting chamber (Hausser Scientific, Horsham, PA).

### Collection of Human Blood Samples

Twenty-eight ml of blood were obtained by venipuncture from mold-exposed patients and unexposed controls after obtaining their consent for research. Blood was withdrawn in four 7 ml tubes containing 0.081 ml of 15% K_3_ EDTA solution (BD Vacutainer, BD, Franklin Lakes, NJ). The samples were coded and shipped to the processing lab in insulated containers at room temperature.

### Pulmonary Function Testing

All mold-exposed patients were requested to undergo methacholine challenges according to a protocol adapted from American Thoracic Society recommendations [44]. All but four of the 33 mold-exposed patients agreed to methacholine challenges. Briefly, prior to administration of methacholine, each patient was first challenged with NaCl. If this dropped their FEV_1_ by 10% from their baseline, the patient response was deemed positive. Mold-exposed patients without a positive response to NaCl were given methacholine at increasing doses and a positive response was defined as an FEV_1_ decrease of 20% or more from baseline following any dose of methacholine up to 16 mg/ml.

### Isolation and Culture of PBMCs

PBMCs were isolated by Ficoll gradient centrifugation, as previously described [45, 46]. Cells were suspended at 10^6^ cells/ml in RPMI 1640 medium supplemented with 1% penicillin-streptomycin, 1% L-glutamine and 10% fetal bovine serum (Invitrogen, Carlsbad, CA).

### *Ex Vivo* Mold and Mycotoxin Exposures of PBMCs

We isolated PBMCs, challenged them *ex vivo*, and measured the cytokines and chemokines that were produced by the cells [46, 47].

We conducted two studies: a “mycotoxin study” where the challenges were either a mycotoxin (SG), an aqueous *S*. *chartarum* extract with a mix of mycotoxins and other antigens (SST), a non-specific toxin (ionomycin [positive control, VWR, Radnor, PA]), or media alone (negative control), each with or without the adjuvant phorbol 12-myristate 13-acetate (PMA, VWR, Radnor, PA); and a “mold spore study” where the challenges were with intact mold spores, a non-specific toxin (phytohemagglutinin [PHA]), or media alone.

#### *S*. *chartarum* mycotoxins and aqueous extracts

Isolated PBMCs were suspended in RPMI at a concentration of 10^6^ cells/ml and mixed with the designated treatment in a microcentrifuge tube, then plated in 96-well plates at a density of 200,000 PBMCs per well. Isolated PBMCs from each patient and control were exposed to SST, SG, ionomycin (1μg/ml), or media, in the presence or absence of PMA (1:1000). The mixture was plated in triplicate 200 μl aliquots in 96-well plates, and then incubated at 37°C with 5% CO_2_. We collected cell culture supernatants 18 hours after the exposures began by centrifuging the 96-well plates and transferring supernatants to new 96-well plates, which were then frozen at -80°C.

#### *A niger*, *C*. *herbarum*, and *P*. *chrysogenum* mold spores

PBMCs were cultured in 6-well plates at 10^6^ cells/ml, 3 ml/well. Mold spores were added at two concentrations each: *A*. *niger*, 800 and 4,000 spores/ml; *C*. *herbarum*, 2,000 and 10,000 spores/ml; and *P*. *chrysogenum*, 4,000 and 20,000 spores/ml. These concentrations were five and twenty-five times higher, respectively, than the lowest observed effect level (LOEL): mold spore concentrations that activated extracellular expression of cytokines and chemokines by a statistically significant value (not shown). We added 10 μg/ml phytohemagglutinin (PHA-P, Sigma-Aldrich, St. Louis, MO) to separate plates as a positive control. PHA has high mitogenic activity and induces PBMC proliferation and cytokine secretion [48, 49]. Three replicate plates were used for each condition. Negative control samples contained media only and were used for determining the basal levels of cytokine and chemokine expression.

### Cytokine and Chemokine Assays

At two points during the study, cell culture supernatants were sent as a batch to Eve Technologies (Calgary, Alberta, Canada) for the measurement of 41 cytokines and chemokines (EGF, eotaxin-1, FGF-2, Flt-3L, fractalkine, G-CSF, GM-CSF, GRO(pan), IFNα2, IFN-γ, IL-1α, IL-1β, IL-1ra, IL-2, IL-3, IL-4, IL-5, IL-6, IL-7, IL-8 (CXCL8), IL-9, IL-10, IL-12 p40, IL-12 p70, IL-13, IL-15, IL-17A, IP-10 (CXCL10), MCP-1, MCP-3, MDC, MIP-1α (CCL3), MIP-1β (CCL4), PDGF-AA, PDGF-AB/BB, RANTES, sCD40L, TGF-α, TNF-α, TNF-β, and VEGF-A). The first batch used Milliplex polystyrene beads (Millipore, Billerica, MA) on a Luminex platform. The second batch used a Milliplex magnetic bead panel, also manufactured by Millipore, on a Luminex platform. The change in platform was necessitated by a change in technology and kit availability. Since some of the magnetic beads were more likely to aggregate in the presence of cellular debris, the cell culture supernatants were filtered before analysis. In our statistical analyses, we excluded cytokines and chemokines that were not consistent between polystyrene and magnetic beads in reference samples (p-values < 0.01). We also excluded from the analyses cytokines and chemokines that were not stable over time based on the subset of the nine mold-exposed patients whose repeat blood samples were collected 18 months apart.

Cytokine and chemokine concentrations from cell-culture supernatants from PBMCs exposed to *A*. *niger*, *C*. *herbarum*, and *P*. *chrysogenum* were measured by Luminex xMAP bead array immunoassays using a Bio-Plex 200 fluorescence bead reader (BioRad Laboratories, Hercules, CA). Three panels of antibody-conjugated beads for measuring human inflammatory cytokines (GM-CSF, IL-1β, IL-6, IL-8, TNF-α), Th1/Th2 cytokines (IFN-γ, IL-2, IL-4, IL-5, IL-10) and chemokines (MIP-1α, MIP-1β, MCP-1, eotaxin, RANTES) (BioSource, Camarillo, CA) were used in the assay according to the manufacturers’ instructions.

### Statistical Methods

We performed both single and multiple-marker analyses to identify cytokine and chemokine response signatures for each mold and mycotoxin exposure.

#### Single-marker analysis

For each mold challenge, to examine associations of cytokine and chemokine response levels with case-control status, we performed single-marker analyses using multiple logistic regressions. We log-transformed cytokine response levels in the model. We adjusted for age, gender, ethnicity and smoking status in the models for mycotoxin challenges. We did not adjust for any covariates in the models for whole mold spore challenges due to missing demographic information for some of the subjects. To correct for multiple testing, we estimated false discovery rates (FDR) and defined statistical significance as FDR q-values <0.05, which indicated that no more than 5% of the significant results by p-value <0.05 were considered to be false positive findings [50, 51]. FDR is a common approach to correct for multiple testing in high-throughput screening data, such as microarrays, proteomics and large-scale genotyping studies. In mold-exposed patients, we also explored associations between lung function (methacholine responses) and biomarker (cytokine and chemokine) response levels stratified by mold challenges, as well as associations between clinical symptoms and biomarkers (cytokine and chemokine response levels) by multiple logistic regression models adjusting for age, gender, ethnicity, smoking status and BMI. All of the above analyses were done using R statistical software.

#### Multiple-marker analysis to identify cytokine and chemokine signatures

Multiple-marker analyses were conducted using the support vector machine (SVM) algorithm. Analyzing multiple biomarkers together generally increases the accuracy of disease risk prediction [52]. The SVM approach is a widely used machine learning algorithm for disease prediction and classification with high dimension data [53]. The SVM approach has been used to identify gene expression patterns [54] and biomarker signatures [55] of cancers[56], diabetes [57], and major adverse cardiac events [58], as well as a recent application to classify liver cirrhosis patients from healthy controls by gut microbiota metagenomics [59]. One of the major advantages of applying SVM is that it is an effective classifier without local minima issues that only depends on parsimonious parameters. In addition to linear classification (such as the commonly used logistic regression model), SVM can efficiently perform non-linear statistical classification, which is an important feature when the relationship between predictors and outcomes are not linear.

To identify the cytokine and chemokine response signatures of mold-exposed patients, we used the heuristic search-SVM [60–63] approach. The model building for the heuristic search-SVM involves four major steps: (1) imputation of missing data, (2) feature (biomarker) selection, (3) classification and (4) cross-validation. The Area Under the Receiver Operating Characteristic (ROC) Curve (AUC) [64] was estimated in the SVM analysis and used to quantify the overall ability of the selected biomarkers to discriminate disease patients from controls. In our study, the AUC was used as the classification performance index to find the best signature (biomarker set) of cytokine and chemokine response levels to differentiate mold-exposed patients from unexposed controls. The SVM analysis was performed using the R svmpath (version 0.953) [65] and the LibSVM (version 3.18) packages [66].

#### Step 1: Imputation of missing data

To minimize the influence of missing biomarkers data in a few samples, we applied a k-Nearest Neighbor (kNN) approach [67] to estimate the missing values for these cytokine response measurements. The selection of the k-nearest cytokines was based on the comparison of cytokine response levels with those of the cytokines of interest available in other samples [67]. In addition, case-control status and mold toxicity challenge information were included in the imputation model to avoid any attenuation of the estimated effects in later analyses [68].

#### Step 2 and Step 3: Feature selection and classification

The feature selection and classification steps were used to identify the optimal combination of cytokine and chemokine responses that can best distinguish cases from controls. This combination of biomarkers can be considered a unique signature of cytokine and chemokine responses in mold-exposed cases. The input features in the SVM model included cytokine responses, chemokine responses, age, gender, ethnicity, and cigarette smoking. Body weight and pulmonary function were not available for unexposed controls and so were not included in the analyses. The feature selection was done by heuristic search [60–63] and performed with a simulated annealing algorithm [69]. This heuristic search algorithm was embedded within the optimization procedure of classification and cross-validation (described below), which increases prediction accuracy [61, 70]. For classification, we used a non-linear SVM classifier with the Gaussian radial basis function kernel. The non-linear SVM classifier mapped all data points into a higher dimensional feature space with non-linear transformation.

#### Step 4: Cross-validation

To reduce the risk of over-fitting and to estimate AUC accuracy, we performed a 10-fold cross-validation [70] 100 times. Each 10-fold cross-validation had a different random seed in order to randomly partition the overall sample into 10 subsets. Since cross-validation produced a potentially different model for each subset of the data, the classification using all observations (i.e., without cross-validation) was displayed for purposes of describing the optimal model.

## Results

### Patient and Control Demographics

The demographics of the mold-exposed patients and unexposed controls for our study are shown in Table 1. The patients were 49.9 ± 8.4 years old (mean ± standard deviation) and the controls were 40.9 ± 15.9 years old. Fifty-two percent of the patients were female and 48% were male, while 29% of the unexposed controls were female and 71% were male. Two patients and four controls were current smokers. Of the patients, 70.4% were Caucasian, 14.8% were Hispanic, 11.1% were Asian, and 3.7% were African-American. Of the controls, 52.9% were Caucasian and 47.1% were African-American.

**Table 1:** Characteristics of subjects who provided blood for *ex vivo* mycotoxin exposures.

		Mold-Exposed Patients (n = 27)	Unexposed Controls (n = 17)
**Age range, years (mean ± SD)**		27–64 (50 ± 8)	18–63 (41 ± 16)
**Gender**			
	Female	14 (52%)	5 (29%)
	Male	13 (48%)	12 (71%)
**Ethnicity**			
	Hispanic	4 (15%)	0 (0%)
	African-American	1 (4%)	8 (47%)
	Asian	3 (11%)	0 (0%)
	Caucasian	19 (70%)	9 (53%)
**Smokers**		2 (7%)	4 (24%)
**Non-smokers**		25 (93%)	13 (76%)
**Methacholine challenge**			Not done
	Normal	16 (66%)	
	Abnormal	8 (33%)	
	Refused	3	

We defined a positive methacholine response as a response to NaCl or any dose of methacholine up to 16 mg/ml leading to an FEV_1_ decrease of 20% or more from baseline.

The patients in the mold spore study ranged from 37–64 years old. Sixty-four percent of the patients were female and 36% were male. One patient was a current smoker and another was a former smoker. Of the patients, 27% were Caucasian, 36% were Hispanic, and 36% were African American.

#### Industrial hygiene

The industrial hygiene consultants tested multiple places within the workplaces including individual offices and commonly used spaces, such as the lobby, front desk area and lounge. They reported measurable levels of *Cladosporium*, *Stachybotrys*, *A*. *niger*, and *P*. *chrysogenum* in multiple places in the building. The full industrial hygiene report is available in S1 Table.

### Mycotoxins Alter Expression of Cytokines and Chemokines by PBMCs

#### Single-marker analysis

After we challenged PBMCs from mold-exposed patients and controls with media (negative control), SST, SG, or ionomycin, with or without PMA, we identified the differences in cytokine and chemokine responses between these groups by running a single-marker analysis that controlled for age, gender, ethnicity, and smoking status. The majority of the cytokine and chemokine responses with q<0.05 (false discovery rate q-value correcting for multiple testing, q<0.05 corresponding to p<0.021) were observed in response to SG or SST alone. When PMA was used as an adjuvant with SST, SG or ionomycin, it blunted the differences between mold-exposed patients and controls, and PMA alone caused marked cytokine and chemokine responses. The following cytokines and chemokines were significantly higher in response to both SG and SST in mold-exposed patients compared to controls: eotaxin, INF-α, IL-1α, IL-12 p40, IL-12 p70, IP-10, PDGF-AA, TNF-β, and VEGF (q<0.05 in Table 2). Notably, none of these cytokines or chemokines were significantly higher in cases when PBMCs were exposed to media alone.

**Table 2:** Cytokine and chemokine concentrations in PBMC cultures exposed to Satratoxin G (SG), *Stachybotrys* Spore Toxin (SST), or Ionomycin *ex vivo*.

			Mold-Exposed Patients	Unexposed Controls		Patients/ Controls
	PBMCs Exposure	Cytokine or Chemokine	Adjusted mean ± SEM	Adjusted mean ± SEM	p-value	Fold change
**Mold specific challenge**	**SG**	**EGF (pg/ml)**	23 ± 2	13 ± 3	0.0058	1.8
		**Eotaxin (pg/ml)**	6.2 ± 0.8	0.9 ± 0.9	5.38E-05	6.7
		**INF-α (pg/ml)**	28.0 ± 4.4	2.3 ± 5.2	0.00036	12.2
		**IL-1α (pg/ml)**	3.0 ± 0.6	0.2 ± 0.7	0.0023	18.8
		**IL-8 (ng/ml)**	5.4 ± 0.9	1.7 ± 1.1	0.014	3.2
		**IL-12 p40 (pg/ml)**	22.8 ± 3.0	0.02 ± 3.5	5.24E-06	1137
		**IL-12 p70 (pg/ml)**	1.61 ± 0.23	0.40 ± 0.27	0.0013	4.0
		**IP-10 (pg/ml)**	7.4 ± 0.7	1.6 ± 0.8	4.95E-07	4.7
		**MDC (pg/ml)**	27.9 ± 2.8	2.9 ± 3.3	1.71E-07	9.6
		**PDGF-AA (pg/ml)**	222 ± 51	34 ± 60.	0.020	6.6
		**TNF-β (pg/ml)**	2.76 ± 0.26	0.67 ± 0.31	2.27E-06	4.1
		**VEGF (pg/ml)**	20.3 ± 2.1	4.2 ± 2.5	5.88E-06	4.8
	**SST**	**Eotaxin (pg/ml)**	6.3 ± 0.8	1.8 ± 0.9	0.00029	3.6
		**IFN-α (pg/ml)**	41.0 ± 4.6	4.7 ± 5.5	3.70E-06	8.8
		**IL-1α (pg/ml)**	2.64 ± 0.45	0.37 ± 0.53	0.0017	7.2
		**IL-12 p40 (pg/ml)**	22.5 ± 2.8	0.08 ± 3.3	1.43E-06	269
		**IL-12-p70 (pg/ml)**	1.77 ± 0.20	0.44 ± 0.24	9.02E-05	4.0
		**IP-10 (pg/ml)**	5.39 ± 1.03	0.22 ± 1.22	0.0020	24
		**PDGF-AA (pg/ml)**	84 ± 13	29 ±15	0.011	2.8
		**TNF-β (pg/ml)**	2.91 ± 0.27	0.51 ± 0.32	3.11E-07	5.6
		**VEGF (pg/ml)**	16.1 ± 2.8	4.7 ± 3.3	0.011	3.5
**Non-specific challenge**	**Ionomycin**	**IL-1rα (pg/ml)**	133 ± 19	22 ± 22	0.00034	6.0
		**IL-6 (pg/ml)**	90 ± 17	12 ± 19	0.0029	7.5
		**IL-8 (ng/ml)**	6.9 ± 1.1	1.7 ± 1.3	0.0042	4.0
		**IL-10 (pg/ml)**	42 ± 8	2 ± 10	0.0026	21
		**IL-17 (pg/ml)**	9 ± 2	4 ± 3	0.0062	2.3
		**MCP-1 (ng/ml)**	1.58 ± 0.33	0.30 ± 0.38	0.015	5.3
		**MIP-1α (ng/ml)**	4.48 ± 0.78	0.54 ± 0.90	0.0017	8.3
		**MIP-1β (ng/ml)**	0.90 ± 0.16	0.19 ± 0.19	0.0070	4.7
		**TNF-α (pg/ml)**	510 ± 84	124 ± 96	0.0039	4.1
**Negative Control**	**Media**	**IL-8 (ng/ml)**	4.14 ± 0.61	1.37 ± 0.85	0.019	3.0
		**MIP-1α (ng/ml)**	0.30 ± 0.05	0.12 ± 0.06	0.036	2.6
		**MIP-1β (ng/ml)**	0.12 ± 0.017	0.034 ± 0.023	0.011	3.4

Cytokines and chemokines are listed alphabetically for each exposure. Only cytokines or chemokines whose concentrations were significantly different between mold-exposed patients and controls with an FDR q<0.05 in the single marker analysis are shown. Many cytokines and chemokines that did not reach the q<0.05 threshold are not included in the table. Complete data are in S2 Table. Log-transformed case-control correlation adjusted for age, gender, race, and smoking status was used to calculate p-values. Fold changes were calculated from adjusted case and control means.

#### Multiple-marker analysis

**Fig 1:**
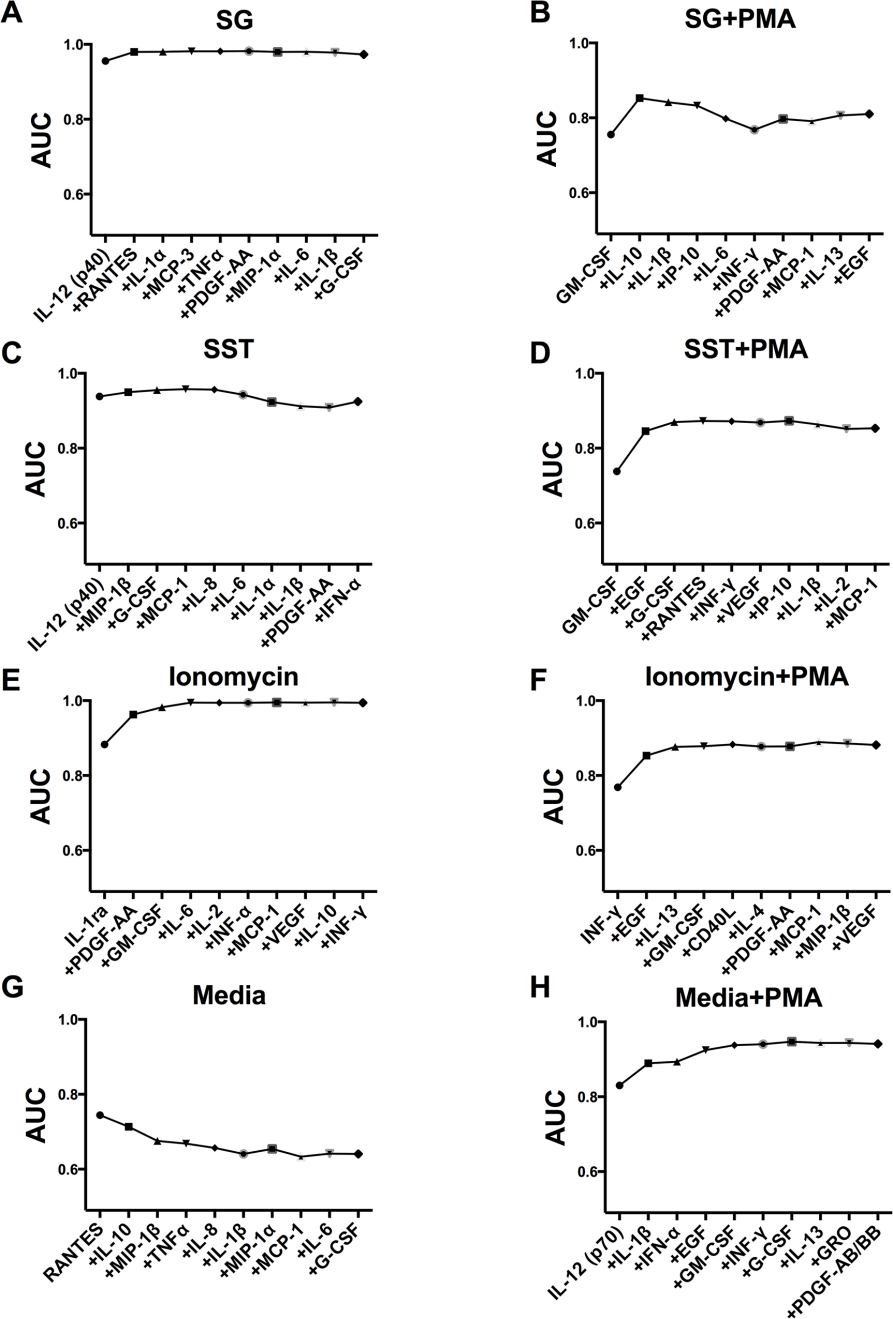
Cytokine and chemokine response signatures and cumulative AUCs from multiple-marker analysis. The estimated cumulative AUCs from SVM analysis are shown to demonstrate the prediction probability of separating mold-exposed patients from controls for combinations of cytokine and chemokine measurements after challenges. The first cytokine or chemokine shown in each graph is the best cytokine or chemokine (among the tested cytokines and chemokines) to differentiate mold-exposed patients from controls after challenge with the listed cellular exposure. Each subsequent cytokine or chemokine adds additional separation (AUC), as depicted on the graph. Each graph shows the top 10 cytokines and chemokines and the resulting AUC when each additional cytokine or chemokine is added to the model. Note that specific cytokines and chemokines on the x-axis generate a toxin-specific signature. A. Satratoxin G (SG), B. SG + phorbol 12-myristate 13-acetate (PMA), C. *S*. *chartarum* spore [mixed] toxins (SST), D. SST + PMA, E. Ionomycin, F. Ionomycin + PMA, G. media (negative control), H. media + PMA.

To identify the cytokine and chemokine response signature in PBMCs of mold-exposed patients after different mycotoxin challenges, we applied the support vector machine (SVM) approach. We found significant response signatures in patient cells challenged with SG, SST and ionomycin (without PMA) (Fig 1).

The area under a receiver operating characteristic (ROC) curve (AUC) ranges between 0.5 and 1.0. A truly insignificant biomarker (one no better at identifying true positives than flipping a coin) has an AUC of 0.5. A perfect set of biomarkers (zero false positives and zero false negatives) has an AUC of 1.00. The higher the AUC, the more accurately the biomarkers have identified (classified) subjects as disease patients versus non-disease controls. These cytokine and chemokine response signatures accurately classified mold-exposed patients from unexposed controls with AUC > 0.97 after controlling for age, gender, ethnicity and smoking status. The cytokine and chemokine response signature of mold-exposed patients was different between SG-exposed PBMCs and SST-exposed PBMCs. The cytokine and chemokine response signature of SG-exposed PBMCs separating mold-exposed patients from unexposed controls comprised six cytokines and chemokines: IL-12 p40, RANTES, IL-1α, MCP-3, TNF-α, and PDGF-AA. The cytokine and chemokine response signature of SST-exposed PBMCs from the mold-exposed group comprised four cytokines and chemokines: IL-12 p40, MIP-1β, G-CSF, and MCP-1. The full cytokine and chemokine data set is available in S2 Table.

### Mold Spores Alter Expression of Cytokines and Chemokines by PBMCs

To evaluate cellular responses to whole mold spores, we challenged PBMCs from mold-exposed patients and unexposed controls with mold spores *ex vivo* and measured the expression of 15 cytokines and chemokines (GM-CSF, IFN-γ, IL1-β, IL-2, IL-4, IL-5, IL-6, IL-8, IL-10, TNF-α, MIP-1β, MCP-1, eotaxin, MIP-1α and RANTES) in cell culture supernatants. Different mold species evoked distinct cytokine and chemokine responses. Seven cytokines and chemokines had significantly different expression profiles between mold-exposed patients and controls in at least one mold challenge (eotaxin, IL1-β, IL-6, IL-8, MCP-1, MIP-1α, and MIP-1β). For *ex vivo* challenges with *P*. *chrysogenum* and *C*. *herbarum*, we observed a significant decrease in multiple cytokine and chemokine responses in mold-exposed patients compared to controls that were not evident with media alone (Table 3). For example, a decrease in the production of eotaxin, IL-8, MCP-1, MIP-1α, and MIP-1β was observed in patient samples challenged with *P*. *chrysogenum* (q<0.05). Lower production of IL-1β, IL-6, MCP-1, and MIP-1α was observed in patient samples challenged with *C*. *herbarum* (q<0.05). In patient samples challenged with *A*. *niger*, there were no significant changes in cytokines or chemokines that were not evident in cells incubated in media alone (q<0.05). Expression levels varied for different cytokines and chemokines but concentrations of most cytokines and chemokines were increased at higher mold doses.

**Table 3:** Cytokine and chemokine concentrations in PBMC cultures exposed to *P*. *chrysogenum*, *C*. *herbarum*, or media alone *ex vivo*.

PBMCs + Exposure		Mold-Exposed Patients	Unexposed Controls		Controls/ Patients
	Cytokine or Chemokine	Mean ± SEM	Mean ± SEM	p-value	Fold Change
***P*. *chrysogenum* (4,000 spores/ml)**	**Eotaxin (pg/ml)**	23 ± 5	84 ± 14	0.00001	3.61
	**IL-8 (ng/ml)**	16.6 ± 4.7	63.7 ± 20.6	0.0026	3.84
	**MCP-1 (ng/ml)**	4.8 ± 1.1	11.8 ± 2.0	0.005	2.42
	**MIP-1α (ng/ml)**	1.4 ± 0.3	13.0 ± 5.5	0.0019	9.45
	**MIP-1β (ng/ml)**	1.7 ± 0.4	7.8 ± 2.6	0.0017	4.57
***C*. *herbarum* (10,000 spores/ml)**	**IL-1β (pg/ml)**	349 ± 83	2585 ± 678	0.008	7.41
	**IL-6 (pg/ml)**	486 ± 127	9596 ± 5304	0.0037	19.7
	**MCP-1 (ng/ml)**	7.8 ± 2.2	2.6 ± 0.6	0.011	0.33
	**MIP-1α (ng/ml)**	6.9 ± 1.5	17.6 ± 3.3	0.006	2.55
**Media**	**Eotaxin (pg/ml)**	16 ± 3	55 ± 7	0.00001	3.36
	**MIP-1α (ng/ml)**	0.11 ± 0.01	0.17 ± 0.01	0.0005	1.60
	**RANTES (ng/ml)**	2.1 ± 0.4	5.2 ± 0.9	0.004	2.45

Cytokines and chemokines are listed alphabetically for each exposure. Only cytokines and chemokines whose concentrations were significantly different between mold-exposed patients and controls with an FDR q<0.05, and whose fold-changes were greater than in media alone are shown. Lower doses of *P*. *chrysogenum* and *C*. *herbarum* and all doses of *A*. *niger* did not cause significant changes (q<0.05) in cytokine and chemokine expression beyond that of the changes in eotaxin, MIP-1α, and RANTES that are visible in media alone. Log-transformed unadjusted case-control correlation was used to calculate p-values. Fold changes were calculated from case and control means.

To determine if differing responses to mold spores in mold-exposed patients and unexposed controls were mold-specific or reflected a change in responsiveness of patient cells, we compared responses to PHA and media alone from mold-exposed patients and controls. There were no significant differences between mold-exposed patients and controls in cytokine or chemokine responses to the PHA challenge (FDR q<0.05, not shown). PBMCs from mold-exposed patients and unexposed controls produced different amounts of eotaxin, MIP-1α, and RANTES in media alone. The magnitude of these differences was more pronounced after *ex vivo* challenges with *C*. *herbarum* and *P*. *chrysogenum*, but not with *A*. *niger* (Table 3). The full cytokine and chemokine data set for the mold spore exposures is available in S3 Table.

### Stratification of Cases

In addition to comparing cases to controls, we took advantage of additional data and stratified cytokine and chemokine responses by methacholine responsiveness and associated symptoms among the mold cases.

#### Asthmatics had a mold-independent cytokine and chemokine response signature

We evaluated the correlation between cytokine and chemokine responses and methacholine test results. In the regression model we used, we adjusted for BMI, age, gender, ethnicity, and smoking status. This adjustment is important because BMI (p = 0.004) and age (p = 0.003) were strongly associated with methacholine results and may directly affect sensitivity to methacholine in a cytokine- and chemokine-independent manner. Though SG and SST alone were the best separators of mold-exposed patients and controls in the single-marker analysis and were excellent separators of mold-exposed patients and controls in the multiple-marker analysis, ionomycin was a better separator of mold-exposed patients with asthma (positive methacholine) from mold-exposed patients without asthma (negative methacholine). We found that MIP-1β, IL-17, TGF-α, and IL-10 were all significantly higher in mold-exposed asthmatics than mold-exposed nonasthmatics (p = 0.007, p = 0.0026, p = 0.0056, and p = 0.0065, respectively; FDR q<0.05) in ionomycin-exposed PBMCs. These four cytokines were likewise significantly higher in mold-exposed nonasthmatics compared to unexposed control subjects (p = 4x10^-5^, p = 6x10^-4^, p = 8x10^-8^, and p = 1x10^-5^, respectively; FDR q<0.05). There were no significant differences in cytokine or chemokine production between mold-exposed nonasthmatics and asthmatics when their PBMCs were exposed to media, SG, SST, *A*. *niger*, *C*. *herbarum* or *P*. *chrysogenum*. This asthma cytokine and chemokine signature was mold-species independent since these differences only became apparent when their cells were exposed to ionomycin *ex vivo*.

#### Cytokine and chemokine responses correlate with clinical symptoms in mold-exposed patients

The mold-exposed patients reported many symptoms consistent with respiratory allergies and asthma. Every patient reported at least one respiratory symptom. More than ⅔ of the mold-exposed patients complained of shortness of breath, coughing, sneezing, runny nose, or nasal congestion. Approximately **½** reported eye itching, burning or tearing and **½** reported wheezing. About ⅓ reported headaches, throat irritation, chronic phlegm production or sinus congestion.

Several cytokine and chemokine responses in SG- and SST-exposed PBMCs were associated with self-reported patient symptoms. Lower levels of CD40L, PDGF-AA, and RANTES after SG challenge were associated with a self-report of head, eye, ear, nose and throat symptoms including itchy, burning or watery eyes; stuffy or runny nose; sneezing; and headaches (p = 0.0003, p = 0.0009, and p = 0.004, respectively; FDR q<0.05). A higher level of human macrophage-derived cytokine (MDC) after SST challenge was also associated with a self-report of these symptoms (p = 0.002, FDR q<0.05). Similarly, a self-report of weight gain is highly correlated with several cytokines and chemokines in response to SST, including INF-α, MDC, and RANTES (p = 0.0002, p = 0.003, and p = 0.01, respectively; FDR q<0.05).

Because 100% of mold-exposed patients reported respiratory symptoms, no correlation with any specific cytokine or chemokine levels in the patient group can be made.

## Discussion

To identify abnormalities associated with indoor environmental mold exposures, we challenged PBMCs from 33 patients who were chronically exposed to molds at their jobsites. When their PBMCs were exposed to mycotoxins, mold extracts, and/or molds *ex vivo*, we found significant differences in the chemokines and cytokines produced. Expression of these proteins differed between the mold-exposed patients and unexposed controls. All the mold-exposed patients reported respiratory symptoms. A third of the mold-exposed patients had abnormal methacholine responsiveness indicative of asthma.

### Cytokine and Chemokine Responses to Mold and Mycotoxin Exposures of PBMCs

From our single-marker analysis, we identified unique cytokine and chemokine response signatures for a history of mold exposure and asthma status. In addition, we used an SVM multiple-marker analysis to identify several mold-specific cytokine and chemokine response signatures in mold-exposed patients. SVM is a popular machine learning algorithm that relies on multiple artificial intelligence algorithms to recognize the patterns/trends in experimental data. It uses recognized patterns to classify and predict in a wide range of scientific applications [53], such as gene expression profiling [54] and biomarker signatures [55] of patients with cancers [71, 72], diabetes [57], or major adverse cardiac events (MACE) [58]. It is an effective classifier without local minima issues and depends only on parsimonious parameters. In addition to linear classification (such as the commonly used logistic regression model), SVM can efficiently perform non-linear statistical classifications, an important feature where the relations between predictors and outcomes are not linear.

The multiple-marker SVM analysis gives an AUC of 0.98 for SG and 0.97 for SST for a particular group of cytokines and chemokines. We identified somewhat different sets of cytokines and chemokines using multiple-marker and the single-marker analyses. Two cytokines or chemokines with significant p-values in the single-marker analysis might be highly correlated. In the multiple-marker analysis, adding a second highly correlated cytokine or chemokine might not add additional separation between the groups versus one of those cytokines or chemokines alone. In the online supplement (S1 Text), we highlight the functions and underlying pathways of the cytokines and chemokines that were identified as potential mediators of inflammatory responses to both SG and SST.

Although each cytokine or chemokine may influence the phenotypes observed, we point out that cytokines and chemokines work together in an orchestrated fashion. It is unlikely that a single cytokine or chemokine will be a perfect biomarker for separating mold-exposed patients and controls. Instead, a pattern of cytokine and chemokine expression must be identified. Quantifying multiple biomarkers increases the accuracy of disease correlations [52]. Most common diseases are caused by the deregulation of complex processes in multiple pathways. Aggregating biomarkers reduces noise and provides more power to detect deregulation of complete functional units, and hence it produces a clearer picture of the underlying pathophysiological process.

Molds may induce persistent changes in inflammatory and immune responses. Epigenetic mechanisms can mediate changes in cytokine and chemokine gene expression and sensitization after chronic mold exposure. Regulatory regions of DNA could be methylated or histones acetylated or deacetylated in response to mold or mycotoxin exposures [73]. This could alter the expression of cytokines and chemokines in response to subsequent stimulation. In addition, memory T cells might activate signaling pathways that trigger cytokine or chemokine release in the presence of mold or mycotoxin challenges.

### Mold and Asthma

We stratified mold-exposed patients by self-reported symptoms as well as by a more objective measure of asthma, methacholine responsiveness. Cytokines and chemokines identified in the initial case-control analysis and the subsequent stratification analysis are more likely to be important mediators of long-term sensitivity to molds. Higher BMI was also correlated with methacholine responsiveness. This is consistent with previously reported correlations between obesity and asthma [74].

Among the mold-exposed patients, only ionomycin challenge differentiated asthmatics from nonasthmatics. Ionomycin challenges of PBMCs may reveal cytokine and chemokine response signatures that differentiate asthmatics from nonasthmatics among mold-exposed patients who have respiratory symptoms. The asthma cytokine and chemokine response signature from ionomycin is distinct from the cytokine and chemokine response signatures from the molds and mycotoxins we tested. Since ionomycin challenges are not specific to mold exposures, ionomycin is a strong candidate for objectively separating asthmatics from nonasthmatics even in a population with other respiratory symptoms. These results should be validated in a larger clinical study.

Our data are novel in that they suggest alterations in immune and inflammatory system pathways in response to molds. The *ex vivo* ionomycin challenges identified four cytokines, IL-17, IL-10, TGF-α, and MIP-1β, that are elevated in mold-exposed asthmatics compared to mold-exposed nonasthmatics and unexposed controls. IL-17 is a lymphokine classically associated with asthma. Th17 cells secrete both it and IL-10 to balance protective and pathologic T-cell responses to multiple pathogens, including molds [75]. Increased levels of IL-10 limit the inflammatory response to pathogens. IL-10 may play a role in down-regulating some proinflammatory cytokines in asthmatic patients with chronic mold exposure [76]. TGF-α is a member of the epidermal growth factor family. It plays a critical role in airway remodeling and mucus production in asthma [77]. TGF-α may exacerbate the respiratory symptoms in asthmatics with chronic mold exposure. MIP-1β (CCL4) is a neutrophil chemoattractant that contributes to both early and late phase asthma responses [78].

The asthmatic response to ionomycin could be the result of differences in sub-populations of PBMCs in asthmatics and nonasthmatics or might be the result of epigenetic changes during asthma development in genes regulating cytokine responses. This result should be validated in a larger population and further mechanistic studies would be required to clarify why the asthmatic patients are more responsive to ionomycin.

Moldy environments may contribute to upper respiratory symptoms even in the absence of IgE-mediated allergic sensitivity [79]. Our results support a link between respiratory illness and human cellular immune and inflammatory responses that have been previously reported [80, 81]. When PBMCs were challenged with mycotoxins or molds, the different patterns of cytokine and chemokine expression observed in mold-exposed patients versus controls was consistent with an activation of inflammatory cells and an increase in cytokine and chemokine production both *in vivo* [28, 82–85] and in cell cultures [37–39, 86–88].

We observed different cytokine and chemokine response signatures after challenges with different mycotoxins or mold species. This specificity may explain differences in cytokine and chemokine expression patterns in other studies [89]. These different signatures support our view that chronic exposures to mold induce altered immune system responses to subsequent mold challenges in a mycotoxin- and mold-specific manner. Because similar cytokine and chemokine responses could be caused by other inflammatory or immune responsive agents such as other allergens or bacteria [90] and we don’t have industrial hygiene reports from the buildings where unexposed control subjects work, a larger clinical study that better characterizes the environment where control subjects work will be required to fully validate a cellular *ex vivo* test that can be used to characterize human health effects from exposures to individual molds. In addition, a future clinical study should include other common respiratory exposures that can lead to cytokine and chemokine release to establish whether these cytokine and chemokine signatures are unique to specific molds.

We demonstrated for the first time that chronic exposures to molds alter cytokine and chemokine responses of PBMCs to specific mold or mycotoxin challenges. Inhibited or enhanced production of cytokines in mold-exposed patients may reflect elements of both sensitization and tolerance. Inhibition of production of some cytokines and chemokines in mold-exposed patients may be analogous to the suppression of inflammatory responses observed in PBMCs from allergen-sensitized subjects [91]. Mold toxins may suppress the immune system through a balance of cytotoxicity and altered Th1/Th2 balance. The alteration of immune responses due to chronic mold exposures may also adversely affect the ability of the immune system to fight infections and other environmental challenges. This may explain patient complaints of concurrent susceptibility to infectious organisms and enhanced responses to chemical irritants. Some changes we observed with *ex vivo* exposure to *S*. *chartarum* mycotoxins were consistent with sensitization. Prior exposures can lead to increases in subsequent inflammatory responses [92].

### Future Studies

Our data are consistent with studies that emphasize the prevention of mold exposures as one way to reduce the incidence and severity of asthma [93]. Since many molds have an adverse impact on respiratory health, it is vital to reduce mold contamination in indoor environments. Especially when there is water intrusion-related mold growth, we should aggressively remediate these contaminated environments as soon as possible to minimize associated health risks. Given the ubiquitous nature of mold spores and their ability to remain dormant and survive in isolated spaces, remediation and renovation of some contaminated buildings may be inadequate and total replacement may be required. Studies of the effectiveness of various remediation strategies are needed.

Mold-induced changes in chemokine and cytokine release may suggest a review of best practices for the diagnosis and treatment of asthma and other respiratory illnesses. For example, current management of patients with mold-related illnesses often involves oral, parenteral or inhalation administration of corticosteroid-based medications that act by suppressing the immune system. However, the underlying illness of asthma, when induced or aggravated by molds, is accompanied by both increases and decreases in immune and inflammatory pathways, as shown by our findings. Perhaps a goal of treatment should include the restoration of normal immune system functioning, in which case steroid-based therapies should be reassessed.

Four of the mold-exposed patients in this study transferred to uncontaminated worksites six months or more before their blood samples were collected. It is important to document to what extent mold-related immune system responses persist after mold exposures cease. This phenomenon may serve as a basic etiologic mechanism in the development of chronic respiratory illnesses such as asthma. Future studies should include a larger number of subjects who have histories of mold exposures but who are no longer being actively exposed.

The results of this study provide guidance for an expanded clinical study to examine these findings in larger population groups. Such a study should measure mold-specific IgE and pulmonary function in all mold-exposed patients and healthy controls. In parallel, a full industrial hygiene assessment is necessary for all patient and control workplaces and homes since we know that mold exposures are not all or nothing, but rather a continuum. A future study might also be designed to assess the contribution of mold exposures to the initiation and aggravation of pediatric and adult asthma.

## Conclusions

We demonstrate for the first time that chronic exposures to environmental molds are associated with changes in cytokine and chemokine production by isolated PBMCs in response to specific mold or mycotoxin challenges. *Ex vivo* exposures of PBMCs to molds or mycotoxins can differentiate mold-exposed patients from unexposed controls. This strategy may be applicable to other inflammation-related environmental toxins.

## Supporting Information

S1 Table Full Industrial Hygiene Report. (PDF)

S2 Table Full cytokine and chemokine data set for *ex vivo* mycotoxin exposures. (XLSX)

S3 Table Full cytokine and chemokine data set for *ex vivo* mold spore exposures. (XLSX)

S1 Text Detailed descriptions of the possible cytokine and chemokine pathways altered by *S*. *chartarum* exposure. (DOCX)
