# Supplementary material for: Environmental Mold and Mycotoxin Exposures Elicit Specific Cytokine and Chemokine Responses
Source: PLoS One. 2015 May 26;10(5):e0126926. doi: 10.1371/journal.pone.0126926 (PMC4444319; doi:10.1371/journal.pone.0126926)
Supplement: S1 Table — (PDF) [file pone.0126926.s001.pdf]

[redacted]

# Table VIIIA

## Total Fungal Spore (Non-Cultured) Ambient Air Sample Results

[redacted]

**Results reported in spores per cubic meter of air (spores/m$^3$)**

| Sample Number | 04-0046-0809-NV1 | 04-0046-0809-NV2 | 04-0046-0809-NV3 | 04-0046-0809-NV4 |
|---|---|---|---|---|
| **Start Time** | 08:54 | 09:24 | 09:47 | 10:08 |
| **Sample Duration** | 5 minutes | 5 minutes | 5 minutes | 5 minutes |
| **Sample Location** | Outdoor control; back parking lot | Lobby (Before HVAC Activation) | [redacted] office (Before HVAC Activation) | Front desk area (Before HVAC Activation) |
| Alternaria | 13 | 347 | 187 | 120 |
| Arthrinium | | | | |
| Ascospores | 107 | 427 | 53 | |
| Aureobasidium | | 13 | | |
| Basidiospores | 213 | 960 | 320 | 320 |
| Beltrania | | | | |
| Bipolaris/Drechslera group | 13 | 27 | | 40 |
| Botrytis | | | | |
| Chaetomium | 27 | 13 | 13 | 13 |
| Cladosporium | 1,010 | 2,190 | 2,350 | 1,010 |
| Curvularia | 27 | 13 | | 27 |
| Epicoccum | 27 | 53 | 40 | 13 |
| Fusarium | | | | |
| Myrothecium | | | 13 | |
| Nigrospora | | | | |
| Oidium | 80 | 80 | 13 | |
| Other brown | 53 | 53 | 53 | |
| Penicillium/Aspergillus types | 907 | 3,790 | 44,500 | 9,070 |
| Pithomyces | | | | 13 |
| Rusts | | 13 | | |
| Scopulariopsis | | | 13 | |
| Smuts, Periconia, Myxomycetes | 67 | 960 | 853 | 627 |
| Stachybotrys | | 13 | | 13 |
| Stemphylium | | | 13 | 13 |
| Torula | | | 13 | 27 |
| Trichocladium | | | | |
| Ulocladium | | | | |
| Zygomycetes | | | | |
| **TOTAL** | **2,544** | **8,952** | **48,434** | **11,306** |

**Notes:**

Unless otherwise noted, indoor air samples were collected at approximate center of room. All samples collected five feet above floor/ground.
Total mold counts may not precisely reflect sum of individual mold counts due to rounding.
Samples collected using Zefon Air-o-Cell cassettes.

**Legend:**
N/A: Not applicable

Pacific Project No: [redacted]


Pacific Health & Safety Consulting, Inc.
2192 Martin, Suite 230, Irvine, CA 92612 Phone: 949.253.4065 Fax: 949.253.4071 Email: customerservice@phsc-web.com

[redacted]

# Table VIIIA

## Total Fungal Spore (Non-Cultured) Ambient Air Sample Results

[redacted]

**Results reported in spores per cubic meter of air (spores/m$^3$)**

| **Sample Number** | 04-0046-0809-NV5 | 04-0046-0809-NV6 | 04-0046-0809-NV7 | 04-0046-0809-NV8 |
|---|---|---|---|---|
| **Start Time** | 10:29 | 10:53 | 11:18 | 12:03 |
| **Sample Duration** | 5 minutes | 5 minutes | 5 minutes | 5 minutes |
| **Sample Location** | [redacted] office (Before HVAC Activation) | System control desk (Before HVAC Activation) | Outdoor control; west sidewalk | Lounge (Before HVAC Activation) |
| Alternaria | 80 | 80 | 200 | 27 |
| Arthrinium | | | | |
| Ascospores | 53 | 53 | 2,610 | |
| Aureobasidium | | | | |
| Basidiospores | 267 | 320 | 1,010 | 160 |
| Beltrania | | | | |
| Bipolaris/Drechslera group | 13 | | 27 | |
| Botrytis | | | | |
| Chaetomium | | 27 | 13 | 27 |
| Cladosporium | 1,550 | 2,240 | 6,290 | 907 |
| Curvularia | | | | 13 |
| Epicoccum | 13 | 40 | 80 | |
| Fusarium | | | | |
| Myrothecium | | | | |
| Nigrospora | | | | |
| Oidium | | | 13 | |
| Other brown | | 13 | 13 | |
| Penicillium/Aspergillus types | 11,600 | 11,800 | 9,070 | 11,100 |
| Pithomyces | | | | |
| Rusts | | | | |
| Scopulariopsis | | | | |
| Smuts, Periconia, Myxomycetes | 293 | 267 | 240 | 147 |
| Stachybotrys | 13 | 13 | 40 | |
| Stemphylium | | | 13 | |
| Torula | 13 | | 40 | |
| Trichocladium | | | | |
| Ulocladium | | | 13 | |
| Zygomycetes | | | | |
| **TOTAL** | **13,895** | **14,853** | **19,672** | **12,381** |

**Notes:**

Unless otherwise noted, indoor air samples were collected at approximate center of room. All samples collected five feet above floor/ground.
Total mold counts may not precisely reflect sum of individual mold counts due to rounding.
Samples collected using Zefon Air-o-Cell cassettes.

**Legend:**

N/A: Not applicable

Pacific Project No: [redacted]



Pacific Health & Safety Consulting, Inc.
2192 Martin, Suite 230, Irvine, CA 92612 Phone: 949.253.4065 Fax: 949.253.4071 Email: customerservice@phsc-web.com

[redacted]

# Table VIIIA

## Total Fungal Spore (Non-Cultured) Ambient Air Sample Results

[redacted]

**Results reported in spores per cubic meter of air (spores/m$^3$)**

| **Sample Number** | 04-0046-0809-NV9 | 04-0046-0809-NV10 | 04-0046-0809-NV11 | 04-0046-0809-NV12 |
|---|---|---|---|---|
| **Start Time** | 12:26 | 12:48 | 13:12 | 13:33 |
| **Sample Duration** | 5 minutes | 5 minutes | 5 minutes | 5 minutes |
| **Sample Location** | Computer / Billing room (Before HVAC Activation) | Outdoor control; back parking lot | [redacted] office (Before HVAC Activation) | Maintenance room (Before HVAC Activation) |
| Alternaria | 27 | 133 | 147 | 27 |
| Arthrinium | | | | |
| Ascospores | | | 53 | 53 |
| Aureobasidium | | | | |
| Basidiospores | 107 | 107 | 533 | 267 |
| Beltrania | | | 13 | |
| Bipolaris/Drechslera group | 27 | 40 | 13 | 13 |
| Botrytis | | | | |
| Chaetomium | | 13 | 27 | |
| Cladosporium | 480 | 1,280 | 1,870 | 1,650 |
| Curvularia | | 27 | 13 | 27 |
| Epicoccum | 13 | 80 | 13 | |
| Fusarium | | | | |
| Myrothecium | | | | |
| Nigrospora | | | | |
| Oidium | | 67 | | 13 |
| Other brown | 13 | 53 | 120 | 53 |
| Penicillium/Aspergillus types | 3,570 | 373 | 1,390 | 2,240 |
| Pithomyces | | | | |
| Rusts | | | | |
| Scopulariopsis | | 13 | | |
| Smuts, Periconia, Myxomycetes | 40 | 160 | 747 | 200 |
| Stachybotrys | | 13 | 27 | |
| Stemphylium | | 13 | 13 | |
| Torula | | | | 13 |
| Trichocladium | | | | |
| Ulocladium | | | | |
| Zygomycetes | | | | |
| **TOTAL** | **4,277** | **2,372** | **4,979** | **4,556** |

**Notes:**

Unless otherwise noted, indoor air samples were collected at approximate center of room. All samples collected five feet above floor/ground.
Total mold counts may not precisely reflect sum of individual mold counts due to rounding.
Samples collected using Zefon Air-o-Cell cassettes.

**Legend:**
N/A: Not applicable



Pacific Health & Safety Consulting, Inc.
2192 Martin, Suite 230, Irvine, CA 92612 Phone: 949.253.4065 Fax: 949.253.4071 Email: customerservice@phsc-web.com

[redacted]

# Table VIIIA

## Total Fungal Spore (Non-Cultured) Ambient Air Sample Results

[redacted]

**Results reported in spores per cubic meter of air (spores/m$^3$)**

| **Sample Number** | 04-0046-0809-NV13 | 04-0046-0809-NV14 | 04-0046-0809-NV15 | 04-0046-0809-NV16 |
|---|---|---|---|---|
| **Start Time** | 14:10 | 14:35 | 14:53 | 15:13 |
| **Sample Duration** | 5 minutes | 5 minutes | 5 minutes | 5 minutes |
| **Sample Location** | Outdoor control; back parking lot | Lobby (After HVAC Activation) | [redacted] office (After HVAC Activation) | Front desk (After HVAC Activation) |
| Alternaria | 40 | 40 | 40 | 40 |
| Arthrinium | | | | |
| Ascospores | 53 | 53 | | 53 |
| Aureobasidium | | | | |
| Basidiospores | 107 | 213 | 160 | 160 |
| Beltrania | | | | |
| Bipolaris/Drechslera group | | 13 | | |
| Botrytis | | | | |
| Chaetomium | | 13 | | 13 |
| Cladosporium | 4,320 | 3,630 | 2,880 | 1,330 |
| Curvularia | | | | 13 |
| Epicoccum | 27 | 13 | | 13 |
| Fusarium | | | | |
| Myrothecium | | 13 | 13 | |
| Nigrospora | | | | |
| Oidium | 27 | | | 13 |
| Other brown | 53 | 13 | | 53 |
| Penicillium/Aspergillus types | 800 | 7,200 | 13,700 | 3,840 |
| Pithomyces | | | | |
| Rusts | | | | |
| Scopulariopsis | | | | |
| Smuts, Periconia, Myxomycetes | 93 | 147 | 53 | 133 |
| Stachybotrys | | | | |
| Stemphylium | | | | |
| Torula | | | | |
| Trichocladium | | | | |
| Ulocladium | | | | |
| Zygomycetes | | | | |
| **TOTAL** | **5,520** | **11,348** | **16,846** | **5,661** |

**Notes:**

Unless otherwise noted, Indoor air samples were collected at approximate center of room. All samples collected five feet above floor/ground.
Total mold counts may not precisely reflect sum of individual mold counts due to rounding.
Samples collected using Zefon Air-o-Cell cassettes.

**Legend:**

N/A: Not applicable

Pacific Project No: [redacted]



Pacific Health & Safety Consulting, Inc.
2192 Martin, Suite 230, Irvine, CA 92612 Phone: 949.253.4065 Fax: 949.253.4071 Email: customerservice@phsc-web.com

[redacted]

# Table VIIIA

[redacted]

**Results reported in spores per cubic meter of air (spores/m³)**

| Sample Number | 04-0046-0809-NV21 | 04-0046-0809-NV22 | 04-0046-0809-NV23 | 04-0046-0809-NV24 |
|---|---|---|---|---|
| **Start Time** | 16:46 | 17:04 | 17:22 | 17:45 |
| **Sample Duration** | 5 minutes | 5 minutes | 5 minutes | 5 minutes |
| **Sample Location** | Computer / Billing room (After HVAC Activation) | Mike Blake's office (After HVAC Activation) | Maintenance room (After HVAC Activation) | Outdoor control; back parking lot |
| Alternaria | 13 | 53 | 13 | 40 |
| Arthrinium | | | | |
| Ascospores | | 107 | 160 | 267 |
| Aureobasidium | | | | |
| Basidiospores | 107 | 693 | 107 | 53 |
| Beltrania | | | | |
| Bipolaris/Drechslera group | | | | |
| Botrytis | | | | |
| Chaetomium | | | | |
| Cladosporium | 747 | 1,650 | 1,170 | 3,040 |
| Curvularia | | 13 | | 13 |
| Epicoccum | 13 | 40 | | |
| Fusarium | | | | |
| Myrothecium | | | | |
| Nigrospora | | | | |
| Oidium | 27 | 27 | 13 | 27 |
| Other brown | 13 | 40 | 13 | 13 |
| Penicillium/Aspergillus types | 1,600 | 1,280 | 1,120 | 693 |
| Pithomyces | | | | |
| Rusts | | | | |
| Scopulariopsis | | | | |
| Smuts, Periconia, Myxomycetes | 67 | 347 | 107 | 27 |
| Stachybotrys | | 13 | | |
| Stemphylium | | | | 13 |
| Torula | | | | |
| Trichocladium | | 13 | | |
| Ulocladium | | | | |
| Zygomycetes | | | | |
| **TOTAL** | **2,587** | **4,276** | **2,703** | **4,186** |

**Notes:**

Unless otherwise noted, indoor air samples were collected at approximate center of room. All samples collected five feet above floor/ground.
Total mold counts may not precisely reflect sum of individual mold counts due to rounding.
Samples collected using Zefon Air-o-Cell cassettes.

**Legend:**
N/A: Not applicable

Pacific Project No: [redacted]



Pacific Health & Safety Consulting, Inc.
2192 Martin, Suite 230, Irvine, CA 92612 Phone: 949.253.4065 Fax: 949.253.4071 Email: customerservice@phsc-web.com

[redacted]

# Table VIIIA

## Total Fungal Spore (Non-Cultured) Ambient Air Sample Results

[redacted]

**Results reported in spores per cubic meter of air (spores/m$^3$)**

| **Sample Number** | 04-0046-0809-NV25 | N/A | N/A | N/A |
|---|---|---|---|---|
| **Start Time** | N/A | | | |
| **Sample Duration** | N/A | | | |
| **Sample Location** | Field Blank | | | |
| Alternaria | | | | |
| Arthrinium | | | | |
| Ascospores | | | | |
| Aureobasidium | | | | |
| Basidiospores | | | | |
| Beltrania | | | | |
| Bipolaris/Drechslera group | | | | |
| Botrytis | | | | |
| Chaetomium | | | | |
| Cladosporium | | | | |
| Curvularia | | | | |
| Epicoccum | | | | |
| Fusarium | | | | |
| Myrothecium | | | | |
| Nigrospora | | | | |
| Oidium | | | | |
| Other brown | | | | |
| Penicillium/Aspergillus types | | | | |
| Pithomyces | | | | |
| Rusts | | | | |
| Scopulariopsis | | | | |
| Smuts, Periconia, Myxomycetes | | | | |
| Stachybotrys | | | | |
| Stemphylium | | | | |
| Torula | | | | |
| Trichocladium | | | | |
| Ulocladium | | | | |
| Zygomycetes | | | | |
| **TOTAL** | **None detected** | | | |

**Notes:**

Unless otherwise noted, indoor air samples were collected at approximate center of room. All samples collected five feet above floor/ground.
Total mold counts may not precisely reflect sum of individual mold counts due to rounding.
Samples collected using Zefon Air-o-Cell cassettes.

**Legend:**

N/A: Not applicable

Pacific Project No: [redacted]



Pacific Health & Safety Consulting, Inc.
2192 Martin, Suite 230, Irvine, CA 92612 Phone: 949.253.4065 Fax: 949.253.4071 Email: customerservice@phsc-web.com

[redacted]

# Table IXA

## Viable (Cultured) Fungi Ambient Air Sample Results

[redacted]

**Results reported in colony forming units per cubic meter of air (cfu/m$^3$)**

| **Sampling Location / Activities** | Outdoor control; back parking lot | | Lobby (Before HVAC Activation) | | [redacted] office (Before HVAC Activation) | | Front desk area (Before HVAC Activation) | |
|---|---|---|---|---|---|---|---|---|
| **Sample Number** | 04-0046-0809-M1 | 04-0046-0809-D1 | 04-0046-0809-M2 | 04-0046-0809-D2 | 04-0046-0809-M3 | 04-0046-0809-D3 | 04-0046-0809-M4 | 04-0046-0809-D4 |
| **Sample Media** | MEA | DG18 | MEA | DG18 | MEA | DG18 | MEA | DG18 |
| **Start/Stop Time** | 09:01/09:04 | 08:54/08:57 | 09:31/09:34 | 09:24/09:27 | 09:54/09:57 | 09:47/09:50 | 10:15/10:18 | 10:08/10:11 |
| **Sample Duration** | 3 minutes | 3 minutes | 3 minutes | 3 minutes | 3 minutes | 3 minutes | 3 minutes | 3 minutes |
| Alternaria | | | | | | | | |
| Aspergillus flavus | 12 | 35 | | | | | | |
| Aspergillus fumigatus | 12 | | | | 24 | | | |
| Aspergillus glaucus | | | | 12 | | | | |
| Aspergillus nidulans | 24 | 12 | | | | | | |
| Aspergillus niger | 59 | 59 | 35 | 24 | 35 | 24 | 35 | 12 |
| Aspergillus ochraceus | | | | | | | | |
| Aspergillus sydowii | | 12 | | 12 | 24 | | 71 | 59 |
| Aspergillus terreus | | | | | | | | |
| Aspergillus ustus | | | | | | | | |
| Aspergillus versicolor | | 35 | 530 | 1,300 | 506 | 1,240 | 931 | 1,680 |
| Aureobasidium | | | | | | | 12 | |
| Bipolaris/Drechslera | | | | | | | | |
| Cladosporium | 165 | 165 | 71 | 294 | 12 | 71 | 118 | 12 |
| Epicoccum | | | 12 | | | | | |
| Fusarium | | | | | | | | |
| Non-sporulating fungi | | | | | | | | |
| Paecilomyces | | | | | 24 | 12 | 12 | 12 |
| Penicillium brevicompactum | | | | | | | | |
| Penicillium chrysogenum | | | 47 | | 94 | 130 | 130 | 82 |
| Penicillium citrinum | | | 12 | | | | | |
| Penicillium corylophilum | | | | | | | | |
| Penicillium decumbens | | | 94 | 271 | 188 | 483 | 188 | 400 |
| Penicillium glabrum | | | | | | | | |
| Penicillium italicum | | | | | | | | |
| Penicillium paxilli | | | | 47 | | | | |
| Penicillium purpurogenum | | | | | | | | |
| Penicillium species | 12 | 12 | | | | | | |
| Phoma/Coelomycetes | 59 | | 12 | | | | | |
| Rhizopus | 12 | | | | | | | |
| Ulocladium | | 12 | | | | 12 | 12 | |
| Wallemia | | | 35 | | | | 177 | |
| Yeasts | | | | | | | | |
| **TOTAL** | **355** | **342** | **848** | **1,960** | **907** | **1,972** | **1,686** | **2,257** |

**Notes:** Unless otherwise noted, indoor air samples were collected at approximate center of room. All samples collected approximately five feet above floor/ground. Total mold counts may not precisely reflect sum of individual mold counts due to positive hole correction.

**Legend:**
- DG18: Dichloran-glycerol agar
- MEA: Malt extract agar
- N/A: Not applicable



Pacific Health & Safety Consulting, Inc.
2192 Martin, Suite 230, Irvine, CA 92612 Phone: 949.253.4065 Fax: 949.253.4071 Email: customerservice@phsc-web.com

[redacted]

# Table IXA

## Viable (Cultured) Fungi Ambient Air Sample Results

[redacted]

**Results reported in colony forming units per cubic meter of air (cfu/m$^3$)**

| **Sampling Location / Activities** | [redacted] office (Before HVAC Activation) | | System control desk (Before HVAC Activation) | | Outdoor control; west sidewalk | | Lounge (Before HVAC Activation) | |
|---|---|---|---|---|---|---|---|---|
| **Sample Number** | 04-0046-0809-M5 | 04-0046-0809-D5 | 04-0046-0809-M6 | 04-0046-0809-D6 | 04-0046-0809-M7 | 04-0046-0809-D7 | 04-0046-0809-M8 | 04-0046-0809-D8 |
| **Sample Media** | MEA | DG18 | MEA | DG18 | MEA | DG18 | MEA | DG18 |
| **Start/Stop Time** | 10:37/10:40 | 10:29/10:32 | 10:59/11:02 | 10:53/10:56 | 11:25/11:28 | 11:18/11:21 | 12:11/12:14 | 12:03/12:06 |
| **Sample Duration** | 3 minutes | 3 minutes | 3 minutes | 3 minutes | 3 minutes | 3 minutes | 3 minutes | 3 minutes |
| Alternaria | | | | | | | | |
| Aspergillus flavus | | | | | 12 | | | |
| Aspergillus fumigatus | | | 24 | | | | | |
| Aspergillus glaucus | | | | | | | | 12 |
| Aspergillus nidulans | | | 47 | | | | 35 | |
| Aspergillus niger | | | 12 | 12 | 141 | 153 | 24 | 35 |
| Aspergillus ochraceus | | | | | | | | |
| Aspergillus sydowii | 47 | 82 | | | | | | 12 |
| Aspergillus terreus | | | | | | | | |
| Aspergillus ustus | | | | | | | | |
| Aspergillus versicolor | 506 | 1,300 | 907 | 1,740 | | 12 | 1,200 | 1,480 |
| Aureobasidium | | | | | | | | |
| Bipolaris/Drechslera | | | | | | | | |
| Cladosporium | 35 | 59 | 47 | 165 | 530 | 836 | 118 | 177 |
| Epicoccum | | | | | 71 | | | |
| Fusarium | | | | | | | | |
| Non-sporulating fungi | | | | | | | | |
| Paecilomyces | 12 | | 12 | | | | | |
| Penicillium brevicompactum | | | | | | | | |
| Penicillium chrysogenum | 106 | 141 | 82 | 165 | | | 118 | 106 |
| Penicillium citrinum | | | | | | | | |
| Penicillium corylophilum | | | | | | | | |
| Penicillium decumbens | 177 | 554 | 389 | 318 | | | 130 | 365 |
| Penicillium glabrum | | | | | | | | |
| Penicillium italicum | | | | | | | | |
| Penicillium paxilli | | | | | | | | |
| Penicillium purpurogenum | | | | | | | | |
| Penicillium species | | | 12 | | 12 | 12 | | |
| Phoma/Coelomycetes | | | 12 | | | | 12 | |
| Rhizopus | 12 | | | | | | | |
| Ulocladium | | | | | | | | |
| Wallemia | | | | | | | 35 | |
| Yeasts | | | 12 | | | 12 | | |
| **TOTAL** | **895** | **2,136** | **1,556** | **2,400** | **766** | **1,025** | **1,672** | **2,187** |

**Notes:** Unless otherwise noted, indoor air samples were collected at approximate center of room. All samples collected approximately five feet above floor/ground. Total mold counts may not precisely reflect sum of individual mold counts due to positive hole correction.

**Legend:**

DG18: Dichloran-glycerol agar
MEA: Malt extract agar
N/A: Not applicable

Pacific Project No: [redacted]



Pacific Health & Safety Consulting, Inc.
2192 Martin, Suite 230, Irvine, CA 92612 Phone: 949.253.4065 Fax: 949.253.4071 Email: customerservice@phsc-web.com

# Table IXA

## Viable (Cultured) Fungi Ambient Air Sample Results

[redacted]

**Results reported in colony forming units per cubic meter of air (cfu/m$^3$)**

| Sampling Location / Activities | Computer / Billing room (Before HVAC Activation) | | Outdoor control; back parking lot | | [redacted] office (Before HVAC Activation) | | Maintenance room (Before HVAC Activation) | |
|---|---|---|---|---|---|---|---|---|
| **Sample Number** | 04-0046-0809-M9 | 04-0046-0809-D9 | 04-0046-0809-M10 | 04-0046-0809-D10 | 04-0046-0809-M11 | 04-0046-0809-D11 | 04-0046-0809-M12 | 04-0046-0809-D12 |
| **Sample Media** | MEA | DG18 | MEA | DG18 | MEA | DG18 | MEA | DG18 |
| **Start/Stop Time** | 12:33/12:36 | 12:26/12:29 | 12:55/12:58 | 12:48/12:51 | 13:19/13:22 | 13:12/13:15 | 13:42/13:45 | 13:33/13:36 |
| **Sample Duration** | 3 minutes | 3 minutes | 3 minutes | 3 minutes | 3 minutes | 3 minutes | 3 minutes | 3 minutes |
| Alternaria | | | | | | | | |
| Aspergillus flavus | | | 12 | 12 | | 12 | | |
| Aspergillus fumigatus | 24 | | 12 | | | | 12 | |
| Aspergillus glaucus | | | | | | | | |
| Aspergillus nidulans | | | | 12 | | | 12 | |
| Aspergillus niger | 12 | 47 | 24 | 59 | 59 | 59 | 24 | 47 |
| Aspergillus ochraceus | | | | | | | | |
| Aspergillus sydowii | | 12 | | | | 24 | 35 | 35 |
| Aspergillus terreus | | | | | | | | |
| Aspergillus ustus | | | | | | | | |
| Aspergillus versicolor | 848 | 1,170 | | 24 | 495 | 1,960 | 247 | 766 |
| Aureobasidium | | | | | | | | |
| Bipolaris/Drechslera | | | 12 | | | | | |
| Cladosporium | 24 | 71 | 518 | 495 | 47 | 389 | 177 | 200 |
| Epicoccum | | | 12 | | | | 12 | |
| Fusarium | | | | | | | | |
| Non-sporulating fungi | | | 12 | | | | | |
| Paecilomyces | | | | | 47 | | 24 | 24 |
| Penicillium brevicompactum | | | | | | | | |
| Penicillium chrysogenum | 118 | 71 | | | 35 | 47 | 24 | |
| Penicillium citrinum | | | | | | | | |
| Penicillium corylophilum | | | | | | | | 12 |
| Penicillium decumbens | 118 | 177 | | | 59 | 82 | 35 | 82 |
| Penicillium glabrum | | | 12 | | | | | |
| Penicillium italicum | | | 24 | | | | | |
| Penicillium paxilli | | | | | | | | |
| Penicillium purpurogenum | | | 12 | | | | | |
| Penicillium species | | | | 12 | | | | |
| Phoma/Coelomycetes | | | 24 | 12 | | | 24 | |
| Rhizopus | | | | 12 | 12 | | | |
| Ulocladium | | | | | | | | |
| Wallemia | 118 | | | 12 | | | 82 | 12 |
| Yeasts | | | 12 | 12 | | | | |
| **TOTAL** | **1,262** | **1,548** | **686** | **662** | **754** | **2,573** | **708** | **1,178** |

**Notes:** Unless otherwise noted, indoor air samples were collected at approximate center of room. All samples collected approximately five feet above floor/ground. Total mold counts may not precisely reflect sum of individual mold counts due to positive hole correction.

**Legend:**
- DG18: Dichloran-glycerol agar
- MEA: Malt extract agar
- N/A: Not applicable

Pacific Project No: [redacted]



Pacific Health & Safety Consulting, Inc.
2192 Martin, Suite 230, Irvine, CA 92612 Phone: 949.253.4065 Fax: 949.253.4071 Email: customerservice@phsc-web.com

[redacted]

# Table IXA

## Viable (Cultured) Fungi Ambient Air Sample Results

[redacted]

**Results reported in colony forming units per cubic meter of air (cfu/m$^3$)**

| Sampling Location / Activities | Outdoor control; back parking lot | | Lobby (After HVAC Activation) | | [redacted] office (After HVAC Activation) | | Front desk (After HVAC Activation) | |
|---|---|---|---|---|---|---|---|---|
| **Sample Number** | 04-0046-0809-M13 | 04-0046-0809-D13 | 04-0046-0809-M14 | 04-0046-0809-D14 | 04-0046-0809-M15 | 04-0046-0809-D15 | 04-0046-0809-M16 | 04-0046-0809-D16 |
| **Sample Media** | MEA | DG18 | MEA | DG18 | MEA | DG18 | MEA | DG18 |
| **Start/Stop Time** | 14:17/14:20 | 14:10/14:13 | 14:41/14:44 | 14:35/14:38 | 14:58/15:01 | 14:53/14:56 | 15:20/15:23 | 15:13/15:16 |
| **Sample Duration** | 3 minutes | 3 minutes | 3 minutes | 3 minutes | 3 minutes | 3 minutes | 3 minutes | 3 minutes |
| Alternaria | | | | | | | | |
| Aspergillus flavus | | | | 12 | | | | |
| Aspergillus fumigatus | | | | | | | | |
| Aspergillus glaucus | | | | | | | | |
| Aspergillus nidulans | | | | | | | 12 | |
| Aspergillus niger | 24 | 118 | | | | | | 24 |
| Aspergillus ochraceus | | | | | | | | |
| Aspergillus sydowii | | | | 12 | | 24 | | 12 |
| Aspergillus terreus | | | | | | | | |
| Aspergillus ustus | | | | | | | | |
| Aspergillus versicolor | | | | 259 | | 188 | 59 | 106 |
| Aureobasidium | | | | | | | | |
| Bipolaris/Drechslera | | | | | | | | |
| Cladosporium | 789 | 660 | 59 | 577 | 82 | 389 | 94 | 200 |
| Epicoccum | 35 | | 12 | | | | | |
| Fusarium | | | | | | | | |
| Non-sporulating fungi | | | 12 | | | | | |
| Paecilomyces | | | | | | | | |
| Penicillium brevicompactum | | | | | | | | |
| Penicillium chrysogenum | 24 | 47 | 3,310 | 3,660 | 3,220 | 2,080 | 883 | 1,450 |
| Penicillium citrinum | | | | | | | | |
| Penicillium corylophilum | | | 24 | | 118 | | | |
| Penicillium decumbens | 35 | 12 | | 141 | | 153 | 94 | 71 |
| Penicillium glabrum | | | | | | | | |
| Penicillium italicum | | | | | | | | |
| Penicillium paxilli | | | | | | | | |
| Penicillium purpurogenum | | | | | | | | |
| Penicillium species | | | | | | | | |
| Phoma/Coelomycetes | 24 | | | | | | | 12 |
| Rhizopus | 12 | | | | | | | |
| Ulocladium | | | | | | | | |
| Wallemia | | | | | | | | |
| Yeasts | | | | | | | | |
| **TOTAL** | **943** | **837** | **3,417** | **4,661** | **3,420** | **2,834** | **1,142** | **1,875** |

**Notes:** Unless otherwise noted, indoor air samples were collected at approximate center of room. All samples collected approximately five feet above floor/ground. Total mold counts may not precisely reflect sum of individual mold counts due to positive hole correction.

**Legend:**
- DG18: Dichloran-glycerol agar
- MEA: Malt extract agar
- N/A: Not applicable



Pacific Health & Safety Consulting, Inc.
2192 Martin, Suite 230, Irvine, CA 92612 Phone: 949.253.4065 Fax: 949.253.4071 Email: customerservice@phsc-web.com

# Table IXA

## Viable (Cultured) Fungi Ambient Air Sample Results

[redacted]

**Results reported in colony forming units per cubic meter of air (cfu/m$^3$)**

| **Sampling Location / Activities** | [redacted] office (After HVAC Activation) | | System control desk (After HVAC Activation) | | Lounge (After HVAC Activation) | | Outdoor control; west sidewalk | |
|---|---|---|---|---|---|---|---|---|
| **Sample Number** | 04-0046-0809-M17 | 04-0046-0809-D17 | 04-0046-0809-M18 | 04-0046-0809-D18 | 04-0046-0809-M19 | 04-0046-0809-D19 | 04-0046-0809-M20 | 04-0046-0809-D20 |
| **Sample Media** | MEA | DG18 | MEA | DG18 | MEA | DG18 | MEA | DG18 |
| **Start/Stop Time** | 15:36/15:39 | 15:30/15:33 | 15:55/15:58 | 15:48/15:51 | 16:11/16:14 | 16:05/16:08 | 16:31/16:34 | 16:36/16:39 |
| **Sample Duration** | 3 minutes | 3 minutes | 3 minutes | 3 minutes | 3 minutes | 3 minutes | 3 minutes | 3 minutes |
| Alternaria | | | | | | | 24 | |
| Aspergillus flavus | | | | | | | | 12 |
| Aspergillus fumigatus | | | | | | | 35 | |
| Aspergillus glaucus | | | | | | | | 12 |
| Aspergillus nidulans | | | | | 12 | | | |
| Aspergillus niger | 12 | | 35 | | | 24 | 165 | 47 |
| Aspergillus ochraceus | | | | | | | | 12 |
| Aspergillus sydowii | | | | | | | | |
| Aspergillus terreus | | | | | | | | 12 |
| Aspergillus ustus | | | | | | | | |
| Aspergillus versicolor | 12 | 106 | 59 | 165 | 130 | 224 | | 12 |
| Aureobasidium | | | | | | | | 24 |
| Bipolaris/Drechslera | | | | | | | | |
| Cladosporium | 82 | 165 | 141 | 47 | 71 | 165 | 1,200 | 1,480 |
| Epicoccum | | | | | | | 24 | |
| Fusarium | | | | | | | | |
| Non-sporulating fungi | | | | | | | | 12 |
| Paecilomyces | | | | | | | | |
| Penicillium brevicompactum | | | | | | | | 12 |
| Penicillium chrysogenum | 1,140 | 1,520 | 660 | 895 | 589 | | 12 | 35 |
| Penicillium citrinum | | | | | | | | |
| Penicillium corylophilum | | | | | | 612 | | |
| Penicillium decumbens | 24 | 118 | 82 | 106 | 59 | | 35 | |
| Penicillium glabrum | | | | | | | | |
| Penicillium italicum | | | | | | | | |
| Penicillium paxilli | | | | | | | | |
| Penicillium purpurogenum | | | | | | | | |
| Penicillium species | | | | | | | 12 | 12 |
| Phoma/Coelomycetes | | | | 12 | | | | 35 |
| Rhizopus | 12 | | | | | | 12 | |
| Ulocladium | | | | | | | | |
| Wallemia | | | | | | | | 12 |
| Yeasts | | | | | | | 12 | |
| **TOTAL** | **1,282** | **1,909** | **977** | **1,225** | **861** | **1,025** | **1,531** | **1,729** |

**Notes:** Unless otherwise noted, indoor air samples were collected at approximate center of room. All samples collected approximately five feet above floor/ground. Total mold counts may not precisely reflect sum of individual mold counts due to positive hole correction.

**Legend:**

- DG18: Dichloran-glycerol agar
- MEA: Malt extract agar
- N/A: Not applicable

Pacific Project No: [redacted]



Pacific Health & Safety Consulting, Inc.
2192 Martin, Suite 230, Irvine, CA 92612 Phone: 949.253.4065 Fax: 949.253.4071 Email: customerservice@phsc-web.com

[redacted]

# Table IXA

## Viable (Cultured) Fungi Ambient Air Sample Results

[redacted]

**Results reported in colony forming units per cubic meter of air (cfu/m$^3$)**

| Sampling Location / Activities | Computer / Billing room (After HVAC Activation) | | [redacted] office (After HVAC Activation) | | Maintenance room (After HVAC Activation) | | Outdoor control; back parking lot | |
|---|---|---|---|---|---|---|---|---|
| **Sample Number** | 04-0046-0809-M21 | 04-0046-0809-D21 | 04-0046-0809-M22 | 04-0046-0809-D22 | 04-0046-0809-M23 | 04-0046-0809-D23 | 04-0046-0809-M24 | 04-0046-0809-D24 |
| **Sample Media** | MEA | DG18 | MEA | DG18 | MEA | DG18 | MEA | DG18 |
| **Start/Stop Time** | 16:51/16:54 | 16:46/16:49 | 17:10/17:13 | 17:04/17:07 | 17:29/17:32 | 17:22/17:25 | 17:51/17:54 | 17:45/17:48 |
| **Sample Duration** | 3 minutes | 3 minutes | 3 minutes | 3 minutes | 3 minutes | 3 minutes | 3 minutes | 3 minutes |
| Alternaria | | | | | | | 12 | |
| Aspergillus flavus | | | | | | | | |
| Aspergillus fumigatus | 24 | 12 | 24 | | 12 | | | |
| Aspergillus glaucus | | | | | | | | |
| Aspergillus nidulans | | | | | | | | |
| Aspergillus niger | | 12 | 24 | 24 | 12 | | 12 | 12 |
| Aspergillus ochraceus | | | | | | | 12 | |
| Aspergillus sydowii | 12 | 12 | | | | | | |
| Aspergillus terreus | | | | | | | | |
| Aspergillus ustus | | | 12 | | | | | |
| Aspergillus versicolor | 188 | 283 | 400 | 660 | 106 | 448 | | 12 |
| Aureobasidium | | 12 | | | | | | |
| Bipolaris/Drechslera | | | 12 | | | | | |
| Cladosporium | 94 | 71 | 224 | 294 | 118 | 165 | 1,240 | 1,300 |
| Epicoccum | 12 | | | | | | 24 | |
| Fusarium | | | | | 35 | | 24 | 24 |
| Non-sporulating fungi | | | | | | | | 12 |
| Paecilomyces | | | | | | | | 12 |
| Penicillium brevicompactum | | | | 24 | | | 47 | |
| Penicillium chrysogenum | 353 | 436 | 247 | 118 | 118 | 259 | | 24 |
| Penicillium citrinum | | | | | | | | |
| Penicillium corylophilum | | | | 35 | | | | |
| Penicillium decumbens | 71 | | 35 | 106 | | | | |
| Penicillium glabrum | | | | | | | | |
| Penicillium italicum | | | | | | | 24 | |
| Penicillium paxilli | | | | | | | | |
| Penicillium purpurogenum | | | | | | | | |
| Penicillium species | | | 12 | | | | | 12 |
| Phoma/Coelomycetes | | | | 12 | | | 24 | 47 |
| Rhizopus | | | | | | | | |
| Ulocladium | | | | | | | | |
| Wallemia | 47 | | 12 | | | | | |
| Yeasts | | | | | 12 | | 12 | 12 |
| **TOTAL** | **801** | **838** | **1,002** | **1,273** | **413** | **872** | **1,431** | **1,467** |

**Notes:** Unless otherwise noted, indoor air samples were collected at approximate center of room. All samples collected approximately five feet above floor/ground. Total mold counts may not precisely reflect sum of individual mold counts due to positive hole correction.

**Legend:**
DG18: Dichloran-glycerol agar
MEA: Malt extract agar
N/A: Not applicable



Pacific Health & Safety Consulting, Inc.
2192 Martin, Suite 230, Irvine, CA 92612 Phone: 949.253.4065 Fax: 949.253.4071 Email: customerservice@phsc-web.com

[redacted]

# Table IXA

## Viable (Cultured) Fungi Ambient Air Sample Results

[redacted]

**Results reported in colony forming units per cubic meter of air (cfu/m$^3$)**

| Sampling Location / Activities | Field Blank | | N/A | | N/A | | N/A | |
|---|---|---|---|---|---|---|---|---|
| **Sample Number** | 04-0046-0809-M25 | 04-0046-0809-D25 | | | | | | |
| **Sample Media** | MEA | DG18 | | | | | | |
| **Start/Stop Time** | N/A | N/A | | | | | | |
| **Sample Duration** | N/A | N/A | | | | | | |
| Alternaria | | | | | | | | |
| Aspergillus flavus | | | | | | | | |
| Aspergillus fumigatus | | | | | | | | |
| Aspergillus glaucus | | | | | | | | |
| Aspergillus nidulans | | | | | | | | |
| Aspergillus niger | | | | | | | | |
| Aspergillus ochraceus | | | | | | | | |
| Aspergillus sydowii | | | | | | | | |
| Aspergillus terreus | | | | | | | | |
| Aspergillus ustus | | | | | | | | |
| Aspergillus versicolor | | | | | | | | |
| Aureobasidium | | | | | | | | |
| Bipolaris/Drechslera | | | | | | | | |
| Cladosporium | | | | | | | | |
| Epicoccum | | | | | | | | |
| Fusarium | | | | | | | | |
| Non-sporulating fungi | | | | | | | | |
| Paecilomyces | | | | | | | | |
| Penicillium brevicompactum | | | | | | | | |
| Penicillium chrysogenum | | | | | | | | |
| Penicillium citrinum | | | | | | | | |
| Penicillium corylophilum | | | | | | | | |
| Penicillium decumbens | | | | | | | | |
| Penicillium glabrum | | | | | | | | |
| Penicillium italicum | | | | | | | | |
| Penicillium paxilli | | | | | | | | |
| Penicillium purpurogenum | | | | | | | | |
| Penicillium species | | | | | | | | |
| Phoma/Coelomycetes | | | | | | | | |
| Rhizopus | | | | | | | | |
| Ulocladium | | | | | | | | |
| Wallemia | | | | | | | | |
| Yeasts | | | | | | | | |
| **TOTAL** | **<12** | **<12** | | | | | | |

**Notes:** Unless otherwise noted, indoor air samples were collected at approximate center of room. All samples collected approximately five feet above floor/ground. Total mold counts may not precisely reflect sum of individual mold counts due to positive hole correction.

**Legend:**
- DG18: Dichloran-glycerol agar
- MEA: Malt extract agar
- N/A: Not applicable



Pacific Health & Safety Consulting, Inc.
2192 Martin, Suite 230, Irvine, CA 92612 Phone: 949.253.4065 Fax: 949.253.4071 Email: customerservice@phsc-web.com
