# Supplementary material for: Environmental Mold and Mycotoxin Exposures Elicit Specific Cytokine and Chemokine Responses
Source: PLoS One. 2015 May 26;10(5):e0126926. doi: 10.1371/journal.pone.0126926 (PMC4444319; doi:10.1371/journal.pone.0126926)
Supplement: S1 Text — (DOCX) [file pone.0126926.s004.docx]

**S1 - Text**

**Environmental mold and mycotoxin exposures elicit specific cytokine and chemokine responses**

Jamie H. Rosenblum-Lichtenstein, Yi-Hsiang Hsu, Igor M. Gavin, Thomas C. Donaghey, Ramon M. Molina, Khristy J. Thompson, Chih-Lin Chi, Bruce S. Gillis, and Joseph D. Brain

**Discussion Supplement**

**Cytokine and Chemokine Responses to Mold and Mycotoxin Exposures**

Here we highlight the function of cytokines and chemokines identified by a single-marker analysis as mediators of inflammatory responses to both SG and SST. Almost all of these cytokines and chemokines were also identified as important in a multiple-marker analysis. Those that were not identified in the multiple-marker analysis were highly correlated with another cytokine or chemokine that was identified in the multiple-marker analysis. We focused on cytokines and chemokines whose expressions were altered by SG and SST. These exposures represented a more specific challenge than ionomycin and the differences between mold-exposed patients and controls after *ex vivo* challenge with SG and SST were highly significant in the single marker analysis. SG, SST and ionomycin achieved similarly high AUCs in the multiple-marker SVM analysis.

**Eotaxin**

Eotaxin is an eosinophil chemoattractant that was expressed in higher levels in the cells of mold-exposed patients after *ex vivo* challenge with both SG and SST. Eotaxin has been shown to enhance airway inflammation in response to *S. chartarum* in mice [1] and may play a similar role in sensitized people.

**INF-α**

After *ex vivo* challenge with SG or SST, INF-α levels were higher in mold-exposed patients versus unexposed controls. INF-α augments neutrophil responses [2] and induces cortisol release [3]. Cortisol release into the blood may inhibit other inflammatory responses in the lungs [4], leading to possible tolerance to subsequent mold exposures .

**IL-1α and IL-6**

IL-1α levels were higher in cells from mold-exposed patients after challenge with SG and SST *ex vivo*. IL-1α is induced in response to tissue damage, and we have previously shown that mice that are more sensitive to *S. chartarum* exposure express higher levels of IL-1α and IL-6 after *S. chartarum* exposure than non-sensitive mice [5]. Several *S. chartarum* mycotoxins, including SG, have been previously shown to induce expression of IL-1α [6]. IL-6 induces differentiation of macrophages, which have been shown to play a role in responding to *S. chartarum* [5]. IL-6 is a pro-inflammatory cytokine and was more highly expressed in mold-exposed patients versus controls after *ex vivo* exposure to SST. IL-6 has been shown to induce IL1-Ra and IL-10 expression [7]. IL-6 was identified as the second most important cytokine in response to SST when all of the cytokines and chemokines are taken in combination. IL-6 has also been identified as an important mediator of responses to *S. chartarum* in other human studies [8].

**IL12 p40 and IL-12 p70**

IL12 p40 is one subunit in the heterodimer IL-12 p70 and IL-12 p70 is the bioactive form [9, 10]. IL12 p40 was identified as the most important separator of mold-exposed patients and controls. Both IL-12 p40 and IL-12 p70 responded with higher levels in mold-exposed patients versus unexposed controls after *ex vivo* challenge with SST or SG. IL-12 p40 acts as an IL-12 antagonist and actively competes with IL-12 p70 [11]. IL-12 p40 may play a role in inhibiting Th1 responses in individuals with a history of chronic mold exposure thereby increasing their risk of developing asthma and allergic responses [12]. Measurement at a single time point may not fully illustrate the complex roles IL-12 p40 and IL-12 p70 play in balancing sensitization and tolerance responses to SG and SST.

**IP-10 and TNF-β**

The PBMCs from mold-exposed patients produced more IP-10 and TNF-β than unexposed controls after *ex vivo* challenge with SG or SST. IP-10 (IFN-γ-inducible protein 10) is a chemokine that plays a role in T cell generation and trafficking [13]. IP-10 is typically seen in Th1 responses and like IL-12 may affect Th1/Th2 balance in response to *S. chartarum*. Despite the classic description of asthma as a Th2 disease, higher levels of IP-10 have been associated with asthma [14]. TNF-β is a product of activated lymphocytes [15]. Like IP-10, TNF-β is associated with Th1 responses in asthmatics [16] and may affect Th1/Th2 balance in response to *S. chartarum* in people with chronic exposure to *S. chartarum*.

**PDGF-AA**

PDGF-AA was expressed in higher levels in the cells of mold-exposed patients after *ex vivo* challenge with both SG and SST. The platelet-derived growth factor (PDGF) AA isoform is produced by macrophages in response to injury and it has a role in tissue remodeling [17]. Differences in PDGF-AA production between mold-exposed patients and controls may indicate differences in tissue remodeling that may affect responses to future exposures.

The biology of these cytokines and chemokines may explain the role they are playing in response to chronic mold exposures.

**References**

1. Leino MS, Alenius HT, Fyhrquist-Vanni N, Wolff HJ, Reijula KE, Hintikka EL, et al. Intranasal exposure to Stachybotrys chartarum enhances airway inflammation in allergic mice. Am J Respir Crit Care Med. 2006;173(5):512-8. Epub 2005/12/03. doi: 10.1164/rccm.200503-466OC. PubMed PMID: 16322647.

2. Pechkovsky DV, Potapnev MP, Zalutskaya OM. Different patterns of cytokine regulation of phagocytosis and bacterial killing by human neutrophils. Int J Antimicrob Agents. 1996;7(1):33-40. PubMed PMID: 18611733.

3. Cardoso E, Arzt E, Coumroglon M, Andrada EC, Andrada JA. Alpha-interferon induces cortisol release by human adrenals in vitro. Int Arch Allergy Appl Immunol. 1990;93(2-3):263-6. PubMed PMID: 2099352.

4. Watterberg KL, Scott SM, Backstrom C, Gifford KL, Cook KL. Links between early adrenal function and respiratory outcome in preterm infants: airway inflammation and patent ductus arteriosus. Pediatrics. 2000;105(2):320-4. PubMed PMID: 10654949.

5. Rosenblum Lichtenstein JH, Molina RM, Donaghey TC, Brain JD. Strain differences influence murine pulmonary responses to Stachybotrys chartarum. Am J Respir Cell Mol Biol. 2006;35(4):415-23. Epub 2006/05/13. doi: 10.1165/rcmb.2005-0483OC. PubMed PMID: 16690987; PubMed Central PMCID: PMC2643262.

6. Islam Z, Harkema JR, Pestka JJ. Satratoxin G from the black mold Stachybotrys chartarum evokes olfactory sensory neuron loss and inflammation in the murine nose and brain. Environ Health Perspect. 2006;114(7):1099-107. Epub 2006/07/13. PubMed PMID: 16835065; PubMed Central PMCID: PMC1513335.

7. Steensberg A, Fischer CP, Keller C, Moller K, Pedersen BK. IL-6 enhances plasma IL-1ra, IL-10, and cortisol in humans. Am J Physiol Endocrinol Metab. 2003;285(2):E433-7. doi: 10.1152/ajpendo.00074.2003. PubMed PMID: 12857678.

8. Nielsen KF, Huttunen K, Hyvarinen A, Andersen B, Jarvis BB, Hirvonen MR. Metabolite profiles of Stachybotrys isolates from water-damaged buildings and their induction of inflammatory mediators and cytotoxicity in macrophages. Mycopathologia. 2002;154(4):201-5. PubMed PMID: 12206322.

9. Kalinski P, Vieira PL, Schuitemaker JH, de Jong EC, Kapsenberg ML. Prostaglandin E(2) is a selective inducer of interleukin-12 p40 (IL-12p40) production and an inhibitor of bioactive IL-12p70 heterodimer. Blood. 2001;97(11):3466-9. PubMed PMID: 11369638.

10. Ruotsalainen M, Hirvonen MR, Hyvarinen A, Teija M, Savolainen K, Nevalainen A. Cytotoxicity, production of reactive oxygen species and cytokines induced by different strains of Stachybotrys sp. from moldy buildings in RAW264.7 macrophages. Environ Toxicol Pharmacol. 1998;6(3):193-9. Epub 1998/11/01. PubMed PMID: 21781894.

11. Gillessen S, Carvajal D, Ling P, Podlaski FJ, Stremlo DL, Familletti PC, et al. Mouse interleukin-12 (IL-12) p40 homodimer: a potent IL-12 antagonist. Eur J Immunol. 1995;25(1):200-6. doi: 10.1002/eji.1830250133. PubMed PMID: 7843232.

12. Wills-Karp M. IL-12/IL-13 axis in allergic asthma. J Allergy Clin Immunol. 2001;107(1):9-18. doi: 10.1067/mai.2001.112265. PubMed PMID: 11149983.

13. Dufour JH, Dziejman M, Liu MT, Leung JH, Lane TE, Luster AD. IFN-gamma-inducible protein 10 (IP-10; CXCL10)-deficient mice reveal a role for IP-10 in effector T cell generation and trafficking. J Immunol. 2002;168(7):3195-204. PubMed PMID: 11907072.

14. Ying S, O'Connor B, Ratoff J, Meng Q, Mallett K, Cousins D, et al. Thymic stromal lymphopoietin expression is increased in asthmatic airways and correlates with expression of Th2-attracting chemokines and disease severity. J Immunol. 2005;174(12):8183-90. PubMed PMID: 15944327.

15. Aggarwal BB, Eessalu TE, Hass PE. Characterization of receptors for human tumour necrosis factor and their regulation by gamma-interferon. Nature. 1985;318(6047):665-7. PubMed PMID: 3001529.

16. Mazzarella G, Bianco A, Catena E, De Palma R, Abbate GF. Th1/Th2 lymphocyte polarization in asthma. Allergy. 2000;55 Suppl 61(s61):6-9. PubMed PMID: 10919498.

17. Raines EW, Dower SK, Ross R. Interleukin-1 mitogenic activity for fibroblasts and smooth muscle cells is due to PDGF-AA. Science. 1989;243(4889):393-6. PubMed PMID: 2783498.
